# Widespread Translational Control of Fibrosis in the Human Heart by RNA-Binding Proteins

**DOI:** 10.1161/CIRCULATIONAHA.119.039596

**Published:** 2019-07-09

**Authors:** Sonia Chothani, Sebastian Schäfer, Eleonora Adami, Sivakumar Viswanathan, Anissa A. Widjaja, Sarah R. Langley, Jessie Tan, Mao Wang, Nicholas M. Quaife, Chee Jian Pua, Giuseppe D’Agostino, Shamini Guna Shekeran, Benjamin L. George, Stella Lim, Elaine Yiqun Cao, Sebastiaan van Heesch, Franziska Witte, Leanne E. Felkin, Eleni G. Christodoulou, Jinrui Dong, Susanne Blachut, Giannino Patone, Paul J.R. Barton, Norbert Hubner, Stuart A. Cook, Owen J.L. Rackham

**Affiliations:** 1 Program in Cardiovascular and Metabolic Disorders, Duke–National University of Singapore Medical School, Singapore (S.C., S.S., E.A., S.V., A.W., S.L., M.W., G.D., S.G.S., B.L.G., S.L., E.Y.C., E.C., J.D., S.A.C., O.J.L.R.).; 2 National Heart Centre Singapore, Singapore (S.S., S.L., J.T., C.J.P., S.A.C.).; 3 Cardiovascular and Metabolic Sciences, Max Delbrück Center for Molecular Medicine in the Helmholtz Association, Berlin, Germany (E.A., S.v.H., F.W., S.B., G.P., N.H.).; 4 German Centre for Cardiovascular Research, partner site Berlin, Germany (N.H.).; 5 Charité-Universitätsmedizin, Berlin, Germany (N.H.).; 6 Berlin Institute of Health, Germany (N.H.).; 7 National Heart and Lung Institute, Imperial College London, United Kingdom (N.M.Q., L.E.F., P.J.R.B., S.A.C.).; 8 Medical Research Council-London Institute of Medical Sciences, Hammersmith Hospital Campus, United Kingdom (N.M.Q, S.A.C.).; 9 Cardiovascular Research Centre, Royal Brompton and Harefield National Health Serfice Trust, London, United Kingdom (N.M.Q, P.J.R.B.).

**Keywords:** dilated cardiomyopathy, fibrosis, ribosome profiling, RNA-binding proteins, TGF-beta1

## Abstract

Fibrosis is a common pathology in many cardiac disorders and is driven by the activation of resident fibroblasts. The global posttranscriptional mechanisms underlying fibroblast-to-myofibroblast conversion in the heart have not been explored.

**Methods:**

Genome-wide changes of RNA transcription and translation during human cardiac fibroblast activation were monitored with RNA sequencing and ribosome profiling. We then used RNA-binding protein-based analyses to identify translational regulators of fibrogenic genes. The integration with cardiac ribosome occupancy levels of 30 dilated cardiomyopathy patients demonstrates that these posttranscriptional mechanisms are also active in the diseased fibrotic human heart.

**Results:**

We generated nucleotide-resolution translatome data during the transforming growth factor β1-driven cellular transition of human cardiac fibroblasts to myofibroblasts. This identified dynamic changes of RNA transcription and translation at several time points during the fibrotic response, revealing transient and early-responder genes. Remarkably, about one-third of all changes in gene expression in activated fibroblasts are subject to translational regulation, and dynamic variation in ribosome occupancy affects protein abundance independent of RNA levels. Targets of RNA-binding proteins were strongly enriched in posttranscriptionally regulated genes, suggesting genes such as *MBNL2* can act as translational activators or repressors. Ribosome occupancy in the hearts of patients with dilated cardiomyopathy suggested the same posttranscriptional regulatory network was underlying cardiac fibrosis. Key network hubs include RNA-binding proteins such as Pumilio RNA binding family member 2 (*PUM2*) and Quaking (*QKI*) that work in concert to regulate the translation of target transcripts in human diseased hearts. Furthermore, silencing of both *PUM2* and *QKI* inhibits the transition of fibroblasts toward profibrotic myofibroblasts in response to transforming growth factor β1.

**Conclusions:**

We reveal widespread translational effects of transforming growth factor β1 and define novel posttranscriptional regulatory networks that control the fibroblast-to-myofibroblast transition. These networks are active in human heart disease, and silencing of hub genes limits fibroblast activation. Our findings show the central importance of translational control in fibrosis and highlight novel pathogenic mechanisms in heart failure.

## Abstract 2

Supplemental Digital Content is available in the text.

**Clinical Perspective**

**What Is New?**

- We show that a third of all genes involved in the transforming growth factor β1–driven fibrotic response of cardiac fibroblasts are regulated at the translational level, independent of RNA transcript abundance.
- This is the first study to investigate the overall importance of translational regulatory networks in myocardial fibrosis.
- We identify RNA binding proteins such as Quaking or Pumilio RNA binding family member 2 as regulatory hubs for gene translation in the fibrotic hearts of dilated cardiomyopathy patients.
- Knockdown of RNA-binding proteins, Pumilio RNA binding family member 2 and Quaking, inhibits the activation of fibroblasts with transforming growth factor β1 in vitro.

**What Are the Clinical Implications?**

- There are currently no specific therapies for myocardial fibrosis which predict both electrical and pump failure of the heart and this remains poorly understood.
- We identify previously unappreciated genes under translational control as novel candidates for disease biology and as potential therapeutic targets.
- Critical fibrosis factors impact cellular phenotypes at the protein level which cannot be appreciated using bulk or single cell RNA-sequencing approaches.
- RNA binding proteins are central to the fibrosis response and represent unexplored gene expression regulators and potential diagnostic or therapeutic targets.

Cardiac remodeling, heart failure, and arrhythmia syndromes are frequently associated with fibrosis, which is a common late-stage pathology in many human diseases.^1^ Cardiac fibrosis is seen in numerous cardiac conditions, including atrial fibrillation,^2^ hypertrophic cardiomyopathy,^3^ dilated cardiomyopathy (DCM),^4^ and heart failure with preserved ejection fraction.^5^ Fibrosis of the heart is driven primarily by the activation of resident fibroblasts.^6,7^ A better understanding of the molecular mechanisms underlying fibroblast activation is of great importance for the development of novel antifibrotic therapies.^8^

While various cues initiate the cellular conversion of fibroblasts to myofibroblasts, TGFβ1 (transforming growth factor β1) is considered a master regulator.^9^ However, antifibrotic therapeutic approaches based on TGFβ1 inhibition have side effects due to the pleiotropic roles of this cytokine, especially in cancer and inflammation.^10,11^ Thus, unraveling the fibroblast-specific footprint of TGFβ1 signaling is an important step toward the identification of novel downstream drivers of cardiac disease. While RNA expression changes via TGFβ1-induced SMAD (small mothers against decapentaplegic) signaling have been studied previously,^12,13^ the independent impact of TGFβ1 on RNA translation remains unknown.

To address this gap in knowledge, we profiled genome-wide RNA transcription and translation levels^14^ in human primary cardiac fibroblasts at several time points after TGFβ1 stimulation. A tailored computational analysis^15^ identified posttranscriptional regulatory networks underlying fibroblasts activation. To corroborate our findings in an independent and disease-relevant context, we performed ribosome profiling of cardiac samples from patients with DCM and found much of the network was active in disease. Finally, silencing of key regulatory hubs from these networks was enough to limit the progression of TGFβ1-treated fibroblasts toward the myofibroblast state. This integrative approach provides a detailed perspective on posttranscriptional regulatory hubs in human heart disease.

## Methods

### Data and Software Availability

The raw data are available on the gene expression omnibus (GEO submission: GSE131112, GSE123018, GSE131111). Transcription and translation levels of individual genes are provided as a web resource: http://ribo.ddnetbio.com.

### Human Primary Fibroblast Culture

Human primary atrial fibroblasts were prepared from atrial biopsies of patients undergoing coronary artery bypass grafting in keeping with local guidelines (Singhealth Centralized Institutional Review Board 2013/103/C and 2018/2543) and cultured as described previously (Table I in the online-only Data Supplement).^13^ All the experiments were carried out at low cell passage (<4). In all experiments, cells were starved in serum-free DMEM for 16 hours prior to TGFβ1 stimulation. Stimulated fibroblasts were compared to unstimulated fibroblasts that had been grown for the same duration under the same conditions (serum-free DMEM), but without the stimuli.

### Immunostaining, Operetta High-Content Imaging, and Confocal Microscopy

Operetta phenotyping assay was performed as described previously.^13^ Briefly, atrial fibroblasts were seeded in 96-well black CellCarrier plates (PerkinElmer) at a density of 10^4^ cells/well. Following TGFβ1 stimulation, cells were fixed in 4% paraformaldehyde and permeabilized with 0.1% Triton X-100, and nonspecific sites were blocked with 0.5% BSA and 0.1% Tween-20 in PBS. Cells were incubated overnight (4°C) with primary antibodies (Table II in the online-only Data Supplement), followed by incubation with the appropriate secondary antibodies and counterstained with DAPI (4’,6-diamidino-2-phenylindole, dihydrochloride). Each condition was imaged from duplicate wells (7 fields/well) using Operetta high-content imaging system 1483 (PerkinElmer). The quantification of α-smooth muscle actin (ACTA2; ACTA2^+ve^) cells and fluorescence intensity per area of collagen I and periostin were performed using Harmony v3.5.2 and Columbus 2.7.1 (PerkinElmer). Immunostaining for confocal microscopy was conducted as previously described.^16^ Atrial fibroblasts were cultured in 8-well chamber slides and fixed with 4% formaldehyde. After permeabilization with 0.1% Triton X-100, cells were washed and blocked in 1% BSA in PBS for 30 minutes. Cells were incubated overnight at 4°C with the primary antibodies (Table II in the online-only Data Supplement). After washing and incubation with secondary antibodies for 30 minutes at room temperature, slides were mounted with ProLong gold antifade. Images were acquired using a confocal laser scanning microscope (LSM710, Zeiss).

### ELISA

The amount of MMP2 and TIMP-1 in equal volume of cell culture media was quantified using Total MMP2 (R&D Systems) and TIMP-1 Quantikine ELISA kit (R&D Systems) as per the manufacturer’s instructions.

### Colorimetric Assay

Quantification of total secreted collagen in the cell culture supernatant was performed using Sirius red collagen detection kit (9062, Chondrex) according to the manufacturer’s protocol.

### Western Blotting

Atrial fibroblasts were washed with ice-cold PBS and solubilized by gentle rocking in radioimmunoprecipitation assay buffer containing protease and phosphatase inhibitors (Roche). Protein concentrations were determined by Bradford assay (Bio-Rad). After centrifugation, equal amounts of protein lysates were separated by SDS-PAGE, transferred to polyvinylidene difluoride membrane, and subjected to immunoblot analysis (Table II in the online-only Data Supplement).

### Statistical Analysis

Statistical analyses of high content imaging were performed using GraphPad Prism software (v6.07). Outliers (ROUT 2%, Prism Software) were removed before analysis. Dunnett’s test was used to calculate multiple testing corrected *P* values for comparison of several time points to baseline.

### Ribosome Profiling and RNA Sequencing

For primary cells, primary human atrial fibroblasts outgrown from cardiac tissue biopsies of 4 patients undergoing coronary artery bypass grafting were expanded to reach 80% confluency in several 10-cm dishes. Cells were stimulated with 5 ng/mL TGFβ1 for 45 minutes, 2 hours, 6 hours, and 24 hours. Per condition, 3 10-cm dishes were employed in order to obtain enough material for ribosome profiling (2 dishes) and RNA sequencing (1 dish).

For heart tissue, as part of a larger consortium effort to characterize the cardiac translatome,^17^ we generated ribosome profiling data of left ventricular tissue samples collected during left ventricular device implantation or cardiac transplantation from patients with end-stage DCM (n=30). We specifically selected these 30 DCM patients as they were obtained from the same site of tissue collection (Cardiovascular Research Centre Biobank at Royal Brompton and Harefield National Health Service Trust) and had previously been subjected to RNA sequencing analysis (30 out of the published 97 DCM patient samples) in order to reduce technical variability and facilitate accurate patient stratification based on the degree of cardiac fibrosis.^18^

Ribosome profiling was performed as previously described.^19^ Briefly, snap-frozen cell pellets or 50 to 100 mg of tissue, previously powdered under liquid nitrogen, was lysed in 1 mL cold lysis buffer (formulation as in TruSeq Ribosome Profile, Illumina) supplemented with 0.1 mg/mL cycloheximide to stabilize ribosomal subunits and prevent postlysis translocation. Homogenized and cleared lysates were then footprinted with Truseq Nuclease (Illumina) according to the manufacturer’s instructions. Ribosomes were purified using Illustra Sephacryl S400 columns (GE Healthcare), and the protected RNA fragments were extracted with a standard phenol:chloroform:isoamylalcohol technique. Following ribosomal RNA removal (Mammalian RiboZero Magnetic Gold, Illumina), sequencing libraries were prepared out of the footprinted RNA. Ribosome profiling libraries were pooled to perform multiplex sequencing on Illumina Hiseq machines.

We used RNA sequencing data for left-ventricle tissue from DCM patients that we had previously published (108 control and 97 DCM patients).^18^ To prepare polyA+ RNA sequencing libraries from primary cardiac fibroblasts, total RNA was extracted with Trizol from 1 10-cm dish per condition. Following cleanup with RNeasy kit (Qiagen), ~500 ng of each sample was further processed with the Truseq Stranded mRNA kit (Illumina). Barcoded RNA sequencing libraries were pooled and sequenced on the Illumina HiSeq platform.

DCM tissue studies complied with UK Human Tissue Act guidelines and were carried out with approval from the Royal Brompton and Harefield local ethical review committee and the National Research Ethics Service Committee South Central, Hampshire B (reference 09/H0504/104).

### Data Processing for RNA Sequencing and Ribosome Profiling

Raw sequencing data were demultiplexed with bcl2fastq V2.19.0.316, and the adaptors were trimmed using Trimmomatic V0.36,^20^ retaining reads longer than 20 nucleotides postclipping. RNA sequencing reads were further clipped with FASTX Toolkit V0.0.14 to 29 nucleotides, to allow comparison directly with ribosome profiling reads. Reads were aligned using bowtie^21^ to known mitochondrial RNA, ribosomal RNA, and transfer RNA sequences (RNACentral,^22^ release 5.0); aligned reads were filtered out to retain only ribosome protected fragments (RPFs). Alignment to the human genome (hg38) was carried out using STAR.^23^ Gene expression was quantified on the coding sequence region using uniquely mapped reads (Ensembl database release GRCh38 v86 combined with additional transcripts from RefSeq GRCh38, downloaded January 2018) with feature counts.^24^ Genes with mean transcripts per million mapped reads (TPM) <1 in either RNA sequencing or ribosome profiling across all conditions were removed prior to downstream analysis. Ribotaper was used to obtain the in-frame reads around the start and stop codon.^25^ These peptidyl-sites (P-sites) were then visualized across samples and genes. Heatmap for the ribosome drop-off was generated using the pheatmap, 1.0.8, R package. Principal component analysis was carried out using the prcomp function in R (Table III in the online-only Data Supplement).

### Detection of Differential Translational-Efficiency Genes Using DESeq2

The calculation of change in translational efficiency (ΔTE) for human fibroblast data was done using an interaction term while accommodating for patient effect in the statistical model within DESeq2 (~ Patient + Time + Sequencing + Time:Sequencing).^26^ This allows for the identification of significant differences between time points that are discordant between sequencing methodologies; ie, for changes in ribosome occupancy that are not explained by changes in RNA abundance. The ΔTE fold change derived with this approach is comparable to traditional TE, but also accounts for variance and level of expression. In addition, the statistical model reveals if ΔTE is statistically significant. Since the RNA and RPF fold changes can be obtained by the same process using DESeq2, the fold changes are also directly comparable with ΔTE. In combination, these 3-fold changes can help predict the regulation status of the gene at the transcriptional and translational levels. Results were combined from the Wald test for each time point and likelihood ratio test across all time points (Table III in the online-only Data Supplement).

### Classification of Differentially Transcribed Genes and Differential Translational-Efficiency Genes Into Regulatory Classes

A gene’s regulation class was first classified using an adjusted *P* value threshold <0.05 for ΔTE, ΔRPF (change in RPFs), and ΔRNA (change in mRNA) combined with the associated direction of change (as shown in Table IV in the online-only Data Supplement). These first level classifications are then combined in order to fully characterize the regulation of each gene. For instance, Forwarded genes are transcriptionally driven genes that have significant ΔRPF and ΔRNA, but do not have a significant ΔTE. Conversely, exclusive genes are regulated only translationally and hence have a significant ΔRPF and ΔTE, but no change on the mRNA levels (ie, ΔRNA not significant). In order to further classify the genes with respect to time, we also performed hierarchical clustering on the log fold changes within each category. For instance, forwarded genes were clustered using the ΔRPF and ΔRNA values, while exclusive genes were clustered using ΔRPF and ΔTE values. Hierarchical clustering was carried out using euclidean distances and the ward.D method in the hclust function and cutreeDynamic with default settings.

### Overrepresentation Analysis

R packages topGO^27^ and KEGGrest^28^ were used to carry out (GO: BP, MF, and KEGG pathways) overrepresentation tests for each gene cluster. Genes that are classified as either differentially transcribed genes (DTG) or differential translation-efficiency genes (DTEG) were used as background. Peak files from eCLIP experiments on ENCODE were downloaded and filtered for 8-fold enrichment and *P* value <10^–5^. Peak files were also downloaded from POSTAR^29^ and used with default filters. For each RNA-binding protein (RBP), the actual number of targets in each gene class or cluster was determined using these peak files. Expected distribution for RBP targets in each group was calculated by randomly selecting gene sets of the same size and quantifying the targets found within the group. This was repeated 100 000 times to obtain an empirical *P* value. These *P* values were further corrected for multiple testing using the Benjamini-Hochberg method. The groups with corrected *P* value <0.05 for an RBP were considered overrepresented for its targets. Z-scores were calculated to evaluate the effect size of this overrepresentation.

### DCM Disease Patient Network Analysis

Spearman ranked correlation was calculated between the RBP’s log_10_(TPM_RPF_) and target’s log_10_(TE) across the patient population (n=30, using all ribosome profiling and RNA sequencing matched samples) with the cor.test R function. A permutation (n=10 000) test was carried out to determine the expected number of correlated pairs (RBP:target) that would be found in a random set. The empirical *P* value was calculated, and Benjamini-Hochberg correction was applied for multiple testing. RBPs correlating with more pairs than random at an adjusted *P* value <5% were selected as network hubs. The network was visualized using Cytoscape.^30^ Spearman ranked correlation was calculated between the RBP’s log_10_(TPM_mRNA_) and fibrosis marker’s log_10_(TPM_mRNA_) with the cor.test R function. To maximize the number of comparisons for the correlation between RBPs and disease severity, we used all 97 patient RNA sequencing data. Patient clustering was carried out using hclust R package in default settings based on marker gene expression levels across all 97 patients. Treecut R package was used to obtain 4 levels of severity in fibrosis. Student *t* test was used to determine significance of the difference between RBP expression in patients with low and high fibrosis severity.

### RNA-Binding Protein Short Interfering RNA Knockdown

For knockdown experiments, cells were transfected using Lipofectamine RNAiMax (Life Technologies), following the manufacturer’s instructions for standard forward transfection (6-well plate format) or reverse transfection (96-well plate format). In both cases, short interfering RNAs (Dharmacon) were used at a final concentration of 25 nM. Reverse transfection was the method of choice for high-throughput transfections to be followed by immunostainings on 96-well plates. Per well of a 96-well plate (96-well black CellCarrier, PerkinElmer), 1 × 10^4^ human cardiac fibroblasts were transfected with 25 nM On-Targetplus short interfering RNAs (Dharmacon) in a medium consisting of serum-free Opti-MEM and DMEM supplemented with FBS (10%), combined in a 1:9 ratio. Twenty-four hours later, media were changed, and cells were cultured in serum free DMEM overnight, before being subjected to TGFβ1 stimulation (5 ng/mL) for 6 hours.

## Results

### Translational Profiling During the Activation of Human Cardiac Fibroblasts

**Figure 1.**
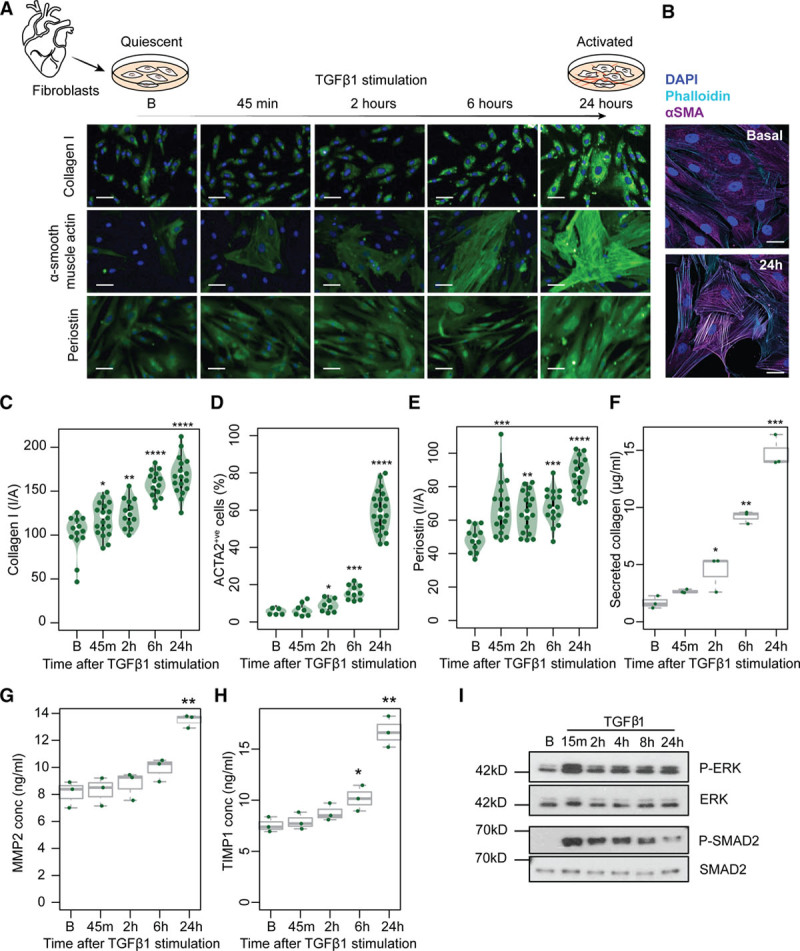
**Time-resolved stimulation of the fibrotic response.** Primary human cardiac fibroblasts were isolated from the atrial biopsies of 4 individuals and stimulated with TGFβ1 (transforming growth factor β1) (5 ng/mL). (**A**) Microscopic images show fibroblasts at 5 timepoints (B, Baseline, 45 minutes, 2 hours, 6 hours, 24 hours after TGFβ1 stimulation) with immunostaining for Collagen I, α-smooth muscle actin (ACTA2) and periostin (POSTN). Scale bar equals 50 µm. (**B**) High-resolution fluorescence imaging with immunostaining of nuclei (DAPI, blue), ACTA2 (purple), and F-actin (phalloidin, cyan) showing TGFβ1 activates fibroblast stress fiber formation. Scale bars indicate 30 µm. (**C** through **E**) Fluorescence was quantified on the Operetta high-content imaging platform after immunostaining for Collagen I (**C**), ACTA2 (**D**), and POSTN (**E**) (28 measurements across 4 wells) and normalized for cell count (**D**) or cell area, I/A, intensity/area (**C** and **E**). Total secreted collagen (**F**), concentration (conc) of MMP2 (**G**), and TIMP-1 (**H**) in the supernatant of TGFβ1-stimulated cardiac fibroblasts (n=3, biologically independent samples) was quantified by Sirius red collagen assay (**F**) and by ELISA (**G** and **H**) respectively. *P* values were determined by one-way ANOVA and corrected for comparisons to the same sample (Baseline) using Dunnett’s test. *P<5×10^-2^, **P<10^–4^, ***P<10^–8^, ****P<2×10^-16^. (**I**) Western blotting of phosphorylated protein (P-) expression of SMAD (small mothers against decapentaplegic)2 and extracellular signal-regulated kinase (ERK) signaling molecules showed rapid activation. B, baseline (0 minutes).

During the fibrotic response, resident fibroblasts become profibrotic myofibroblasts that express *ACTA2* and secrete extracellular matrix proteins such as collagen I and periostin.^31^ To better understand this transition in the human heart, we isolated primary cardiac fibroblasts from atrial biopsies of 4 individuals undergoing coronary artery bypass grafting (Table I in the online-only Data Supplement). TGFβ1 stimulation (5 ng/mL) resulted in a significant increase of ACTA2^+ve^ cells and an upregulation of extracellular matrix-related proteins, indicating activation of fibroblasts within 24 hours (Figure 1a through 1h). This cellular transformation was accompanied by rapid phosphorylation of SMAD as well as extracellular signal-regulated kinase (ERK), which is a key factor in noncanonical signaling pathways and known to regulate posttranscriptional processes (Figure 1i).^13^

**Figure 2.**
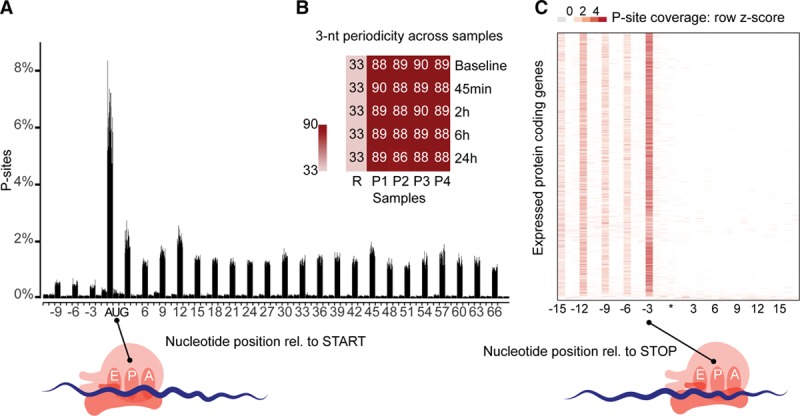
**Ribosome profiling of TGFβ1 (transforming growth factor β1) stimulated primary human cardiac fibroblasts.** (**a**) Sample level periodicity: Distribution of inferred peptidyl-site (P-site) locations (+12 offset) for each sample (4 patients over 5 time points) at annotated translation start sites reveals ribosomes located on the canonical start codon (AUG) and majority of the P-sites downstream of the start codon located in-frame. (**b**) The 3-nt (3-nucleotide)-periodicity for all 20 samples (P1–P4 patients, 5 time points) is >86%, indicating most reads represent actively translating ribosomes. R represents random 3-nt-periodicity of 33%. (**c**) Gene-level periodicity: P-site location across all annotated expressed (transcript per million mapped reads, TPM>1) genes (combined data from 4 patients over 5 time points) shows efficient ribosome drop-off at the canonical stop codon (UGA/UAG/UAA, represented by *). Rel. indicates relative.

To capture a time-resolved snapshot of the molecular changes that underlie the transition of fibroblasts into myofibroblasts, we performed RNA sequencing and Ribo-seq at baseline and at 45 minutes, 2 hours, 6 hours, and 24 hours after TGFβ1 stimulation. Ribosome profiling entails deep sequencing of ribosomal footprints, which are short RNA fragments protected from nuclease treatment by RPFs. RPFs therefore quantify both mRNA abundance and ribosome occupancy of protein-coding genes and as such are a superior proxy for protein levels compared to RNA-seq.^19^ On average we generated ~51 million (RNA sequencing) and ~12 million (ribosome profiling) uniquely mapped reads per sample. Ribosome profiling reads mapped predominantly to the coding sequence and had an average read length of 29 bp (Figure Ia through Id in the online-only Data Supplement), both of which are characteristic of high-quality RPF data.^14^ We then inferred the exact position of the P-site, the site in the ribosome where transfer RNAs recognize their complementary codon, based on RPFs (see Methods). On average, 89% of all P-sites mapped to the coding frame in known genes, indicating that captured ribosomes translate the known reading frame of transcripts (Figure 2a and 2b). Plotting of P-site density around the 3′ location of expressed coding sequence revealed that ribosomes recognized the stop codon and disengaged from RNA transcripts (Figure 2c). High triplet periodicity and a strong dissociation signal reveal the stepwise movement of the ribosome along the coding regions of the transcripts and indicate the positions of actively translating ribosomes being captured at single nucleotide resolution in our ribosome profiling data.

### Dynamic Translational Regulation During the Fibrotic Response

**Figure 3.**
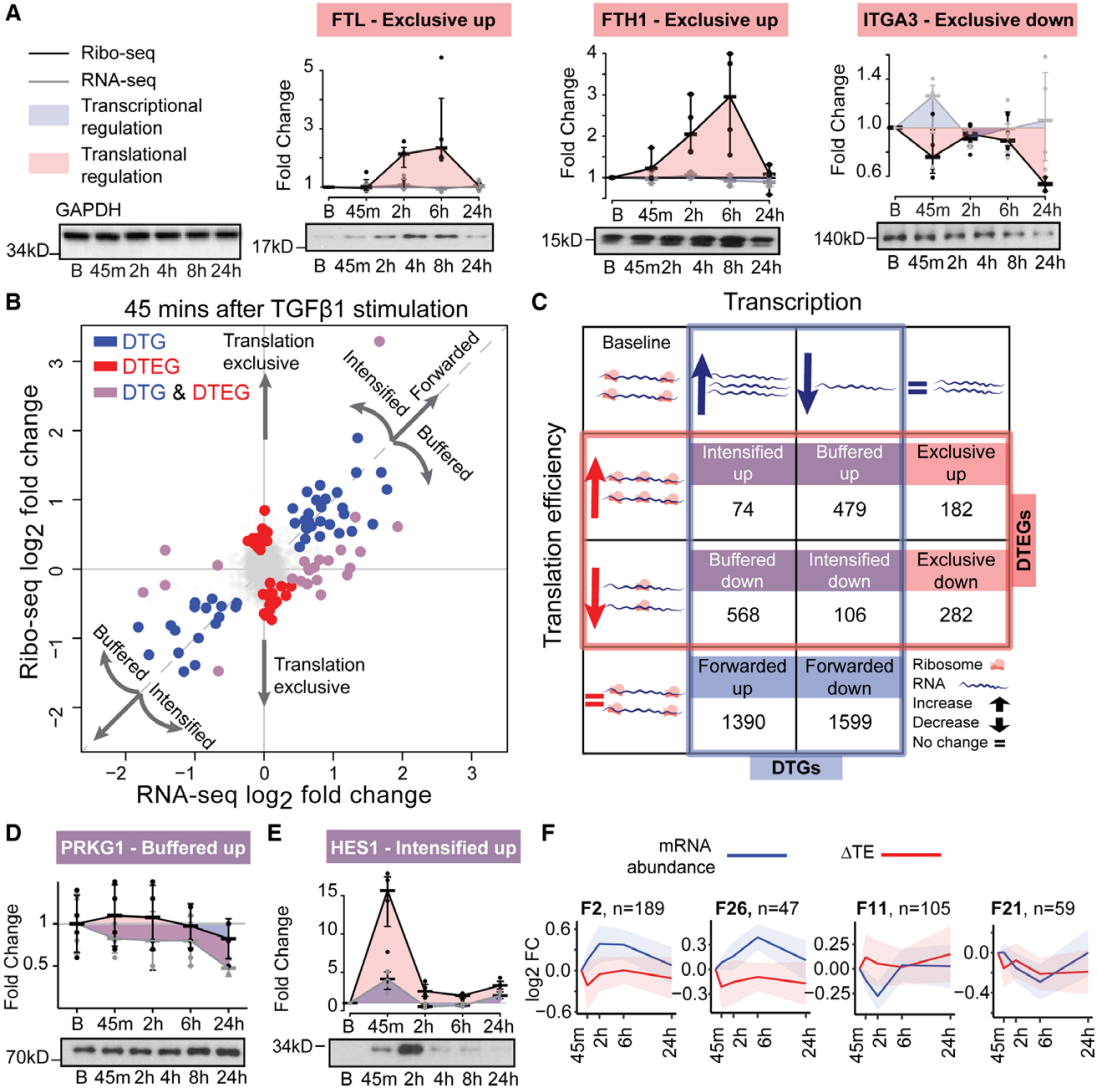
**Genome-wide temporal transcriptional and translational landscape in cardiac fibrosis.** (**a**) Western blots showing ribosome occupancy determining changes in protein levels independent of mRNA changes for translationally exclusive genes, FTL, FTH1, ITGA3. Control: GAPDH. B, basal. (**b**) Log-fold changes in the mRNA and ribosome occupancy at 45 minutes after TGFβ1 (transforming growth factor β1) stimulation. DTEG, differential translational-efficiency genes; DTG, differentially transcribed genes. (**c**) The interplay between DTGs and DTEGs showing several categories of gene expression regulation. Forwarded genes, where the occupancy changes are explained by the mRNA changes; Exclusive, where changes occur exclusively in TE without underlying mRNA changes; Buffered and Intensified, where both the TE and the mRNA are changing. (**d** and **e**) Western blotting of genes detected as buffered, PRKG1 (**d**) and intensified, HES1 (**e**). (**f**)Forwarded gene clusters (F2, F26, F11, F21) with transient changes in expression following TGFβ1 stimulation. FC, fold change; n, number of genes in the cluster.

DTGs can be detected on a genome-wide scale with RNA sequencing alone. Conversely, genes that are translationally regulated between conditions will display a significant change in TE, ie, the genewise ratio between ribosome occupancy and transcript abundance, requiring both RNA sequencing and ribosome profiling for detection. We recently developed an analytical approach (which we refer to as ΔTE) that integrates RNA sequencing and ribosome profiling data to reveal DTEGs.^15^ The output of this approach is a ΔTE value (and associated adjusted *P* value) for each gene describing the log fold-change of TE at each time point. This analysis allowed reliable detection of DTEGs in our data by accounting for the patient-related batch effects (Figure II in the online-only Data Supplement). Using the ΔTE approach, we identified 1691 DTEGs during the fibrotic response. For instance, ribosome occupancy of both *FTL* (Ferritin Light Chain, ΔTE=3.24; P_adj._=2×10^-2^) and *FTH1* (Ferritin Heavy Chain 1, ΔTE=3.12; P_adj._=1x10^-2^) increased significantly upon TGFβ1 stimulation, despite underlying transcript levels remaining the same. Translating ribosomes located on *ITGA3* (Integrin Subunit Alpha 3, ΔTE=-1.9; P_adj._=2×10^-3^) transcripts decreased despite constant levels of RNA. These dynamic and often transient posttranscriptional changes in gene expression were enough to affect protein expression (Figure 3a, Figure IIIa through IIId in the online-only Data). Globally, TGFβ1 signaling had an immediate effect on the ribosome occupancy of 67 genes after 45 minutes (Figure 3b). The most enriched gene ontology term in these early responding genes was “transcription regulator activity” (*P*_adj._=3×10^-3^), suggesting that the following transcriptional response may be modulated by these DTEGs. The impact on translation then gradually decreased at 2 hours and 6 hours but was very pronounced again at 24 hours (File I, and Figure IIIg through IIIj in the online-only Data Supplement).

We detected gradually more DTGs at later time points, up to a total of 4216. Several genes were detected as both DTG and DTEG. This occurs when TGFβ1 affects both RNA levels and TE of the same gene. In order to describe the relationship of this overlap in transcriptional and translational regulation, we categorize each of the DTGs and DTEGs into 1 of 8 regulatory groups (Figure 3c, File II in the online-only Data Supplement). For more than 29% of DTGs, differences in transcription were not forwarded to the translational level but were translationally buffered or intensified. Of these, translational buffering was most prominent, ie, changes in transcript expression detected with RNA sequencing were less pronounced in the ribosome profiling data. This effect can be due to either a less efficient translation of genes whose RNA levels are increasing (568 genes, buffered down) or vice versa, more efficient translation of genes whose RNA levels are decreasing (479 genes, buffered up). Out of these 1047 buffered genes, 419 (231 down, 188 up) had transcriptional regulation that was completely counteracted by translational regulation, resulting in a similar density of translating ribosomes despite underlying TGFβ1-driven transcriptional changes. For example, RNA sequencing suggests a downregulation of the protein kinase *PRKG1* upon TGFβ1 stimulation. However, this effect is not forwarded to the translational level, and thus protein levels do not decrease (Figure 3d, Figure IIIf in the online-only Data Supplement).

For a defined subset of transcripts, RNA expression differences were intensified at the level of translation during the fibrotic response. These genes (n=180) responded even more strongly to TGFβ1 treatment than would be expected from RNA sequencing–based analyses alone. For instance, the concerted upregulation of the transcription factor *HES1* on both the transcriptional and translational levels resulted in a very strong increase in RPFs, which resulted in a profound increase in HES1 protein (Figure 3e, Figure IIIe in the online-only Data Supplement). Interestingly, intensified genes were overrepresented for functions such as “SMAD-protein signal transduction” (*P*_adj._=6.5×10^-3^) and “Regulation of ERK1 and ERK2 cascade” (*P*_adj._=1.4×10^-2^) (File III in the online-only Data Supplement). Overall, these results demonstrate that more than one-third of all gene expression changes during fibroblast activation involve translational regulation and that these changes can affect protein levels.

Transcriptional and translational regulation downstream of TGFβ1 appears to be closely interlinked and tightly regulated over time. To further stratify these effects over time, we performed unsupervised clustering of the temporal profiles of Forwarded, Buffered, Exclusive, and Intensified genes. This revealed 64 distinct regulatory patterns during the fibrotic response (Figure IV in the online-only Data Supplement). The clustering highlights the fact that there are a substantial number of transiently regulated genes during the fibrotic response (Figure 3f). Transient differences in expression would not be apparent when quiescent fibroblasts are compared to myofibroblasts at 24 hours but may be crucial for the cellular transition and therefore important in disease. Transient clusters were predominantly enriched for processes involved in the regulation of gene expression (regulation of RNA metabolic process, 2.5×10^-7^; regulation of transcription, DNA-templated, 1.7×10^-5^), further substantiating their role in fibroblast transformation (File IV in the online-only Data Supplement). We plot RNA sequencing, ribosome profiling, and ΔTE results for any gene of interest at http://ribo.ddnetbio.com and share the values in File I in the online-only Data Supplement.

### RNA-Binding Proteins as Regulators of TGFβ1 Signaling

**Figure 4.**
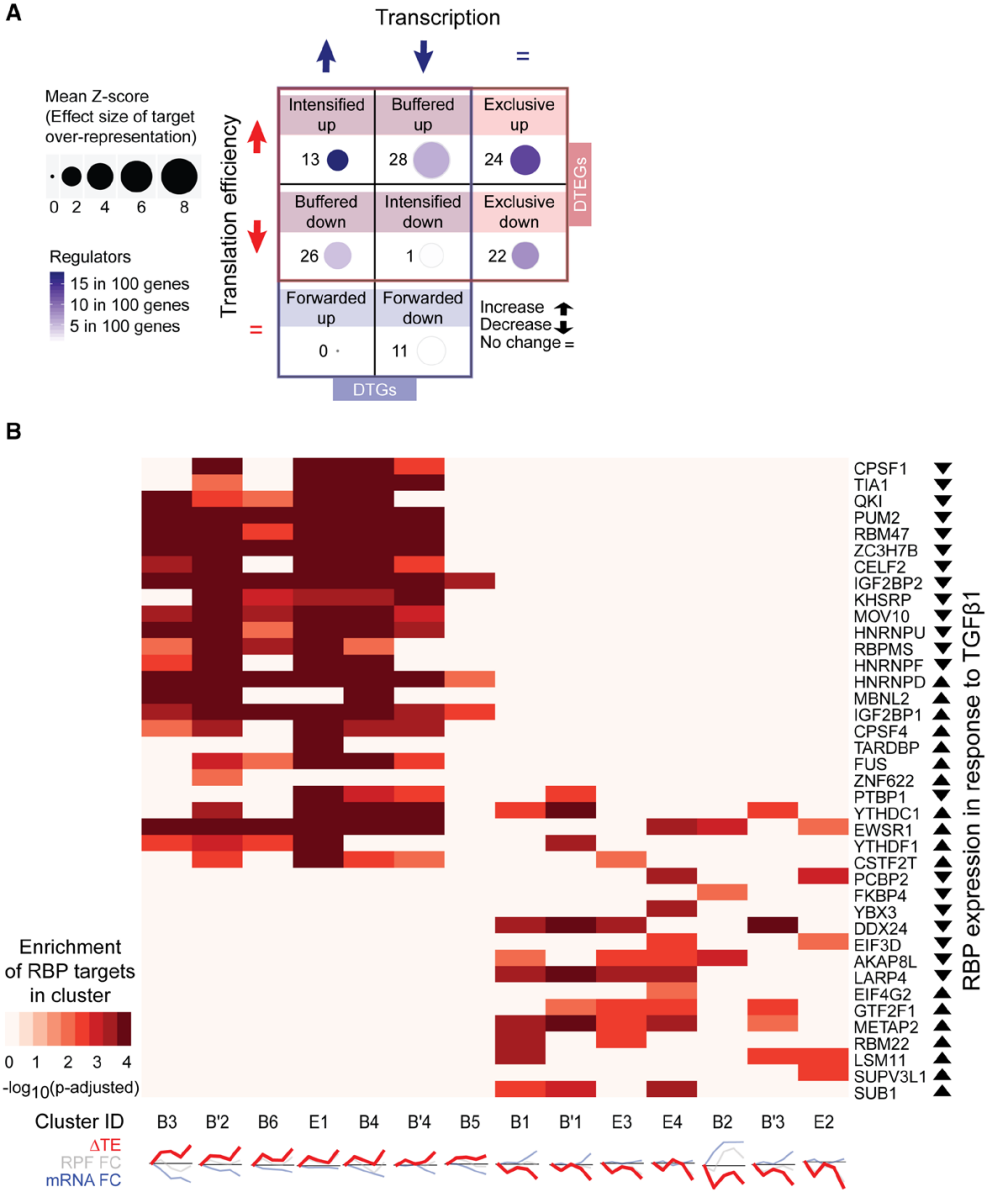
**Posttranscriptional regulators in fibroblast activation.** (**a**) RBP target overrepresentation test in the regulatory groups within DTGs and DTEGs. Z-score is the effect size of overrepresentation. Regulators per member represent the number of RBPs overrepresented per member of the group. (**b**)RBP overrepresentation test (FDR<1%) for regulatory patterns separate translationally activated and repressed clusters. RBP expression in response to TGFβ1 (transforming growth factor β1) is determined using significant RPF changes. RBPs that are not overrepresented for their targets in any translational regulated gene cluster are not shown. Cluster IDs are denoted by their regulatory groups and cluster number. B, buffered; B’, completely buffered (special case); and E, exclusive. Clusters with less than 50 genes, or with no RBP overrepresentation are not shown. DTEG indicates differential translation-efficiency gene; DTG, differentially transcribed genes; FC, fold change; FDR, false discovery rate; RBP, RNA-binding protein; RPF, ribosome protected fragment; and TE, target's translation efficiency.

Having identified different regulatory groups and temporal profiles, we sought to identify potential regulators. It is known that RBPs bind to target transcripts to affect protein production,^19,32^ a number of which been previously linked to heart disease.^33,34^ To identify key RNA-binding proteins during the fibrotic response, we integrated our expression data with more than 200 available transcriptome-wide RBP-RNA binding datasets. We first identified RBPs that were differentially expressed at 45 minutes, 2 hours, 6 hours, and 24 hours after TGFβ1 stimulation in human primary cardiac fibroblasts, revealing 53 differentially expressed RBPs. Next, to construct reliable RBP-target networks, we utilized experimental evidence for RBP-RNA binding derived from eCLIP (n=117), PAR-CLIP (n=58), ^35^ HITS-CLIP (n=23), and iCLIP (n=22) data provided by ENCODE^36^ and POSTAR.^29^ Following this, for each of the differentially expressed RBPs, a permutation test was used to detect whether there was a significant overrepresentation of their targets in each of the 8 regulatory groups defined above, which was the case for 47 RBPs. We found that the targets of these RBPs were predominantly enriched in DTEGs but not in DTGs, which suggests that RBPs shape the fibrotic response mainly through translational regulation and rarely influence transcript levels (Figure 4a). Most targets of individual RBPs were overrepresented in clusters with similar translational regulation profiles (Figure 4b). For instance, QKI targets genes that have an increase in translation, while DDX24 targets genes that have a decrease in translation. These distinct enrichments in unidirectional ΔTE patterns suggest RBPs tend to act either as translational repressors or activators during the fibrotic response.

### RBPs and Their Target Regulatory Networks in the Fibrotic DCM Heart

**Figure 5.**
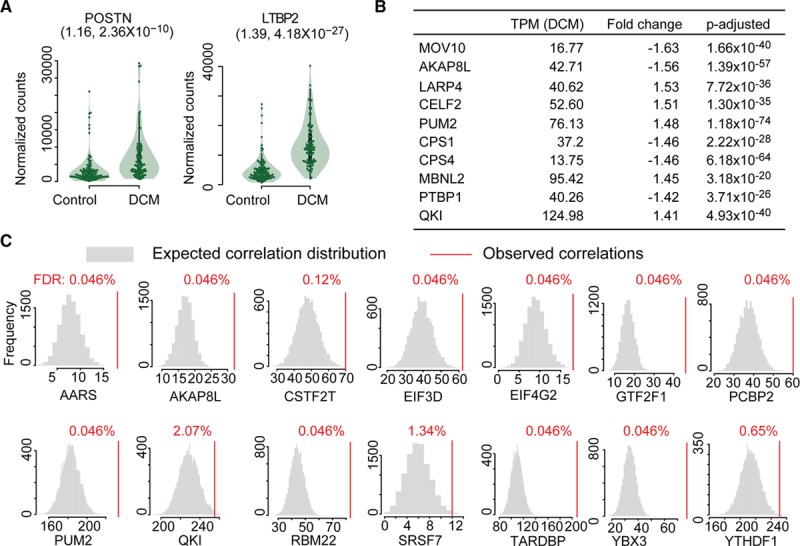
**Posttranscriptional regulators in fibrosis and dilated cardiomyopathy.** (**a**) Periostin (POSTN) and latent TGFβ binding protein 2 (LTBP2) are upregulated in DCM patients. (Fold change, FDR). (**b**) Cardiac expression (transcripts per million mapped reads, TPM) and differential expression (fold change, *P*-adjusted: *P* value corrected by Benjamini-Hochberg) of top 10 RNA binding proteins in DCM patients compared to nondiseased donors. (**c**) RBPs with significantly more correlated targets than expected by chance (RBP RPF vs target TE, FDR values are false discovery rates calculated by the Benjamini-Hochberg method), indicating translational control also in DCM patients. DCM indicates dilated cardiomyopathy; RBP, RNA-binding protein; RPF, ribosome protected fragment; and TE, target's translation efficiency.

To ascertain whether the same translational program was active in disease, we utilized RNA sequencing data from dilated cardiomyopathy and control hearts, which we generated in a previous study (controls=108, DCM patients=97).^18^ Analysis of this data showed upregulation of cardiac fibrosis markers in the hearts of end-stage DCM patients, and expression of these markers was shown to be restricted to cardiac fibroblasts in single-cell RNA sequencing data from the mouse heart (Figure 5a, Figure V in the online-only Data Supplement).^6,37^ Furthermore, 45 of the 47 RBPs identified previously were expressed in DCM hearts (TPM>5), and of these, 22 were differentially expressed between healthy and diseased individuals (fold change≥|1.2|, *P*_adj._≤0.05) (Figure 5b, Figure V in the online-only Data Supplement).

**Figure 6.**
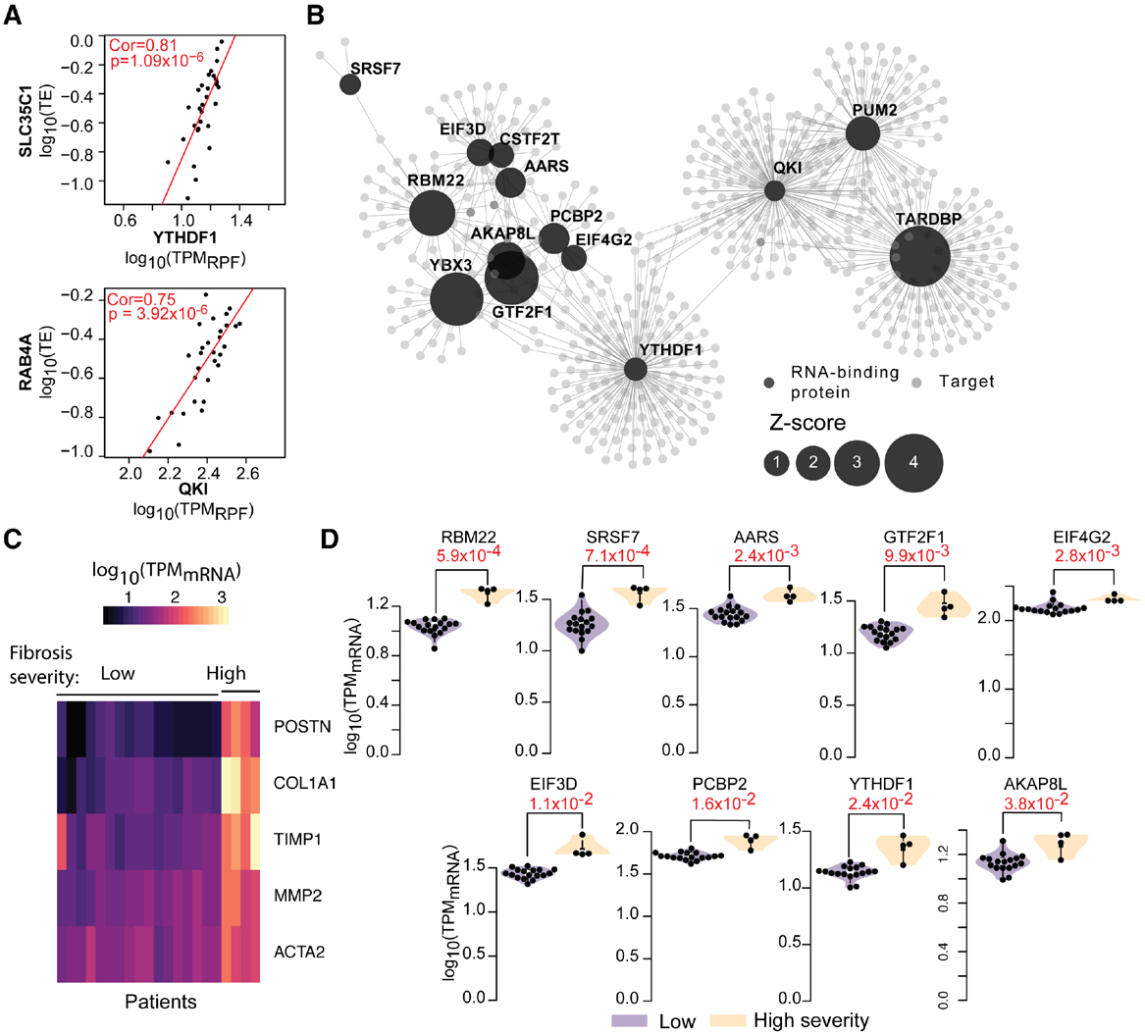
**Posttranscriptional regulatory network in dilated cardiomyopathy.** (**a**) Exemplars of RBP-target pairs correlated in the DCM heart. Cor, Spearman ranked correlation value; p, *P* value for correlation test. (**b**) RBP-target network in disease based on permutation tests ( ρ  ≥ 0.45 for visualization). (**c**) Patient stratification based on severity of fibrosis assessed using marker gene expression (only low and high severity groups shown; full clustering in Figure VIIb in the online-only Data Supplement). (**d**) RBP expression differences between patients with low and high severity of fibrosis. In red, *P* value for 2-tailed *t* test. DCM indicates dilated cardiomyopathy; QKI, Quaking; RBP, RNA-binding protein; RPF, ribosome protected fragment; TE, target's translation efficiency; and TPM, transcripts per million mapped reads.

To assess whether this dysregulation of RBPs at the RNA level also influenced translation in the fibrotic human heart, we performed ribosome profiling of left ventricular tissue collected from a subset of these DCM patients. If the RBPs are truly regulators of ribosome occupancy in fibrosis, then RBP expression levels should correlate with the translational efficiency of their target transcripts. In total, we were able to generate high-depth and high-quality ribosome profiling data of 30 individuals (Figure VI in the online-only Data Supplement; see Methods for details of data generation). We observed a total of 3771 RBP:target pairs correlating significantly in our human heart datasets. Fourteen translational regulators (targeting 926 transcripts) remained significantly correlated with their targets based on permutation analyses (Figures 5c, 6a, and 6b; File V in the online-only Data Supplement), identifying them as regulatory hubs controlling ribosome occupancy both during cardiac fibroblast activation and in the hearts of DCM patients. This substantiates further the influence of RBPs on ribosome occupancy in an independent dataset and provides evidence for posttranscriptional regulatory networks in the human heart.

Modules of this posttranscriptional regulatory network are enriched for distinct biological functions related to actin remodeling and other processes important in fibrosis (File VI in the online-only Data Supplement). One module in the heart is controlled by Pumilio RNA binding family member 2 (PUM2), which is known to drive glial scar formation, a fibrotic and TGFβ1-dependent process in the brain.^38^ Others are regulated by QKI, important in heart development,^39^ or PCBP2, which is known to inhibit cardiac hypertrophy driven by the fibrogenic stimulus angiotensin 2.^40^ Interestingly, we detected a large overlap between targets of regulators, suggesting that the concerted action of several RBPs determines the translational efficiency of bound transcripts. Both QKI and PUM2 appear to cooperate, with more than 21% of their targets (103 genes) overlapping between the RBPs, both of which appear to be acting as translational repressors.

All 14 identified regulatory hubs were correlated significantly on average with 4.5 (out of 5) marker genes such as *ACTA2*, *COL1A1*, or periostin (Figure VIIa in the online-only Data Supplement). Taken together, the dysregulation of these RBPs in the activation of atrial fibroblasts and end-stage DCM patients suggests that these genes regulate translation in the context of cardiac fibrosis. Unsupervised clustering revealed different degrees of fibrogenic marker gene expression (low, moderate, high) in the DCM hearts (Figure 6c, Figure VIIb in the online-only Data Supplement). Nine regulators of the network were significantly elevated in patients with high severity (vs low severity) cardiac fibrosis molecular signature (2-tailed *t* test significance *P* value <0.05, Figure 6d).

**Figure 7.**
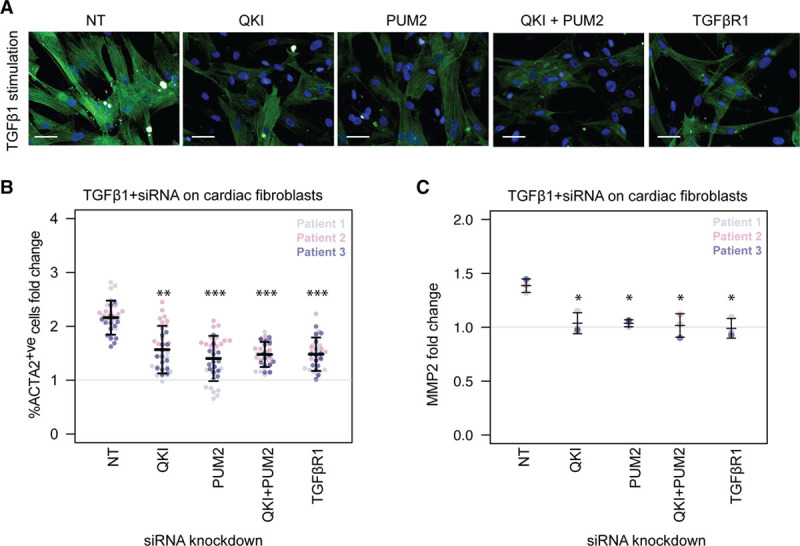
**Effect on fibrotic phenotype after siRNA (short interfering RNA) knockdown of RNA-binding proteins.** Knockdown of Quaking protein (QKI) and Pumilio RNA binding family member 2 (PUM2) followed by stimulation with TGFβ1 (transforming growth factor β1) (5 ng/mL) on fibroblasts from 3 cardiac patients. NT, nontargeting control siRNA. (**a**) Microscopic images show fibroblasts with immunostaining for α-smooth muscle actin (ACTA2). Scale bar equals 50 µm. TGFβ1 stimulation for 6 hours. (**b** and **c**) Fold changes were calculated for %ACTA2^+ve^ cells and MMP2 concentration with respect to NT siRNA in baseline fibroblasts. Fluorescence quantified on the Operetta high-content imaging platform after immunostaining for ACTA2 (28 measurements across 4 wells) and normalized for cell count (**b**). Total concentration of MMP2 in the supernatant of TGFβ1-stimulated cardiac fibroblasts (n=3, biologically independent samples) was quantified by ELISA (**c**). *P* values were determined by 1-way ANOVA and corrected for comparisons to the same sample (NT+TGFβ1) using Dunnett’s test. *P<1.5×10^-3^; **P<2×10^-15^; ***P< 2.22×10^-16^.

In order to ascertain whether some of these RBPs play a driving role in the transition toward profibrotic myofibroblasts in response to TGFβ1, we selected QKI and PUM2 as representative RBPs for further in vitro validation. We targeted these RBPs with short interfering RNAs alone and in combination with TGFβ1 treatment followed by high-content imaging after immunostaining for ACTA2. In the presence of TGFβ1, the knock-down of either QKI or PUM2 alone, as well as in combination, was found to significantly reduce ACTA2 levels in 3 different patients (Figure 7a and 7b, Figure VIII in the online-only Data Supplement). The overall scale of the effect was like that of knocking down TGFβR1, and the combined knock-down appears less variable across patients when compared to knock-downs of QKI or PUM2 alone. To investigate this premise using an orthogonal method, we measured the concentration of MMP2, which is secreted by activated fibroblasts, in cell culture supernatants using ELISA. This revealed a similar reduction in MMP2 secretion (Figure 7c). Taken together, these results demonstrate that a reduction in either QKI or PUM2 or both inhibits fibroblast activation downstream of TGFβ1 stimulation.

## Discussion

These results shed new light on the extent of posttranscriptional regulation in response to extracellular signaling in human disease. Our data suggest that cytokines such as TGFβ1, which are heavily investigated as potential therapeutic targets, directly impact cellular phenotypes via the regulation of translation. While the transcriptional effects of TGFβ1 are well studied,^13^ we show for the first time a genome-wide snapshot of the posttranscriptional effect of the pathway: over one-third of all changing genes are regulated at the translational level. Buffering or intensifying RNA differences and exclusive translational regulation robustly modify protein abundance during the cellular transformation to myofibroblasts. We reveal novel molecular pathways and substantially broaden the scope in the search for urgently needed novel therapeutic targets, which have been missed in previous RNA-based target discovery screens. Discrepancies between transcript and translation levels also have implications for the use of RNA-based biomarkers, which are likely to be less informative than protein-based methods for certain genes.

We found that targets of specific RBPs are significantly enriched in translationally but not transcriptionally regulated genes, revealing their posttranscriptional regulatory footprint during cardiac fibroblast activation. Integration of more than 150 protein–RNA binding data sets with our ΔTE analysis revealed an unprecedented view of the RBP-driven landscape of translational regulation. Some of these identified candidates (*CELF2*,^41^ *PUM2*,^38^ *KHSRP*,^42^ *MBNL2*,^43^ and *LARP4*^44^) are known to repress/activate the translation of single target genes. Our results go beyond this, exposing the extended translational regulatory network of each RBP in the fibroblast to myofibroblast transition. Furthermore, both Quaking (QKI) and MBNL2 have been previously associated with regulating alternative splicing,^45,46^ but our findings suggest that both also regulate the translational efficiency of hundreds of target transcripts during the fibrotic response. This emphasizes the multifunctional role RBPs can play in regulating many posttranscriptional processes such as mRNA splicing, localization, translation, and turnover in parallel.^47^

To further investigate the disease relevance for these networks, we integrated cardiac translatomes of end-stage dilated cardiomyopathy patients. Despite differences between atrial and ventricular fibroblasts,^48^ similarities of the TGFβ1 response across cell types revealed common profibrotic signatures in human cardiac fibrosis (Figure IX in the online-only Data Supplement). We documented highly significant correlation of 14 of these RBPs with the translational efficiency of more than 900 transcripts in the DCM heart, and as such we consider these key regulatory hubs of the diseased cardiac translatome. Regulatory footprints of the networks of these RBP often overlap, suggesting that RBPs act in concert to control the final protein levels of shared targets. This is especially evident with the repressors PUM2 and QKI with more than 21% of their targets overlapping.

Knockdown of PUM2 and QKI blunts the TGFβ1 response in vitro. Translational repression by PUM2 in astrocytes is known to cause astrogliosis and the formation of glial scarring, which is a TGFβ1-dependent process,^38^ and we reveal here the target gene network for PUM2-regulated translation in cardiac scarring. Taken together, this indicates an important role for both QKI and PUM2 in fibrotic cardiac disease.

There are more than 1500 RBPs encoded in the human genome,^49^ but their role in the translation of target mRNAs remains largely unexplored. Just as transcription factors have been targeted therapeutically to modulate transcription, RBPs represent novel diagnostic, prognostic, and therapeutic targets. Translational control may be particularly pertinent in fibroblasts that produce huge amounts of extracellular matrix protein with limited changes in the expression of the correspondingly extracellular matrix transcript.^50^

## Acknowledgments

S.S., S.A.C., N.H., and O.J.L.R. conceived and designed the study. E.A., S.V., A.A.W., J.T., C.J.P., L.E.F., J.D., S.B., M.W., S.L., B.L.G., S.G.S., and G.P. performed in vitro cell culture, cell biology, and molecular biology experiments. S.C., S.S., S.R.L., A.A.W., E.A., G.D., E.Y.C., and E.G.C. analyzed the data with support from N.M.Q., S.v.H., F.W., and P.J.R.B. S.S., S.C., E.A., S.A.C., and O.J.L.R. prepared the manuscript with input from coauthors. The authors would like to acknowledge the technical expertise and support of N.S.J. Ko, J. Schulz, and V. Schneider-Lunitz and the next-generation sequencing Team at National Heart Centre, Singapore.

## Sources of Funding

The research was supported by the National Medical Research Council - Singapore Translational Research investigator awards to S.A.C. (NMRC/STaR/0029/2017), the National Medical Research Council Central Grant to the National Heart Centre Singapore, the Goh Foundation, the Tanoto Foundation, the National Institute for Health Research Cardiovascular Biomedical Research Unit of Royal Brompton and Harefield National Health Service Foundation Trust UK, Heart Research UK (RG2657/17/19), and a grant from the Fondation Leducq. S.S. is supported by the Goh Foundation and Charles Toh Cardiovascular Fellowship. A.A.W. is supported by the National Medical Research Council Young Individual Research Grant (NMRC/OFYIRG/0053/2017). O.J.L.R. is supported by a National Medical Research Council Young Individual Research Grant (NMRC/OFYIRG/0022/2016). N.M.Q. is supported by the Imperial College Academic Health Science Centre.

## Disclosures

S.A.C. and S.S. are coinventors of the patent applications WO2017103108, WO2017103108, WO 2018/109174, and WO 2018/109170. S.A.C. and S.S. are co-ounders and shareholders of Enleofen Bio PTE LTD, a company (which S.A.C. is a director of) that develops antifibrotics. O.J.L.R. is a coinventor of the patent WO/2017/106932 and is a cofounder, shareholder, and director of Cell Mogrify Ltd, a cell therapy company. All other authors declare no competing interests.

## Supplementary Material

## References

[1] Rockey DC, Bell PD, Hill JA (2015). Fibrosis–a common pathway to organ injury and failure.. N Engl J Med.

[2] Burstein B, Nattel S (2008). Atrial fibrosis: mechanisms and clinical relevance in atrial fibrillation.. J Am Coll Cardiol.

[3] Teekakirikul P, Eminaga S, Toka O, Alcalai R, Wang L, Wakimoto H, Nayor M, Konno T, Gorham JM, Wolf CM (2010). Cardiac fibrosis in mice with hypertrophic cardiomyopathy is mediated by non-myocyte proliferation and requires Tgf-β.. J Clin Invest.

[4] Gulati A, Jabbour A, Ismail TF, Guha K, Khwaja J, Raza S, Morarji K, Brown TD, Ismail NA, Dweck MR (2013). Association of fibrosis with mortality and sudden cardiac death in patients with nonischemic dilated cardiomyopathy.. JAMA.

[5] Schelbert EB, Fridman Y, Wong TC, Abu Daya H, Piehler KM, Kadakkal A, Miller CA, Ugander M, Maanja M, Kellman P (2017). Temporal relation between myocardial fibrosis and heart failure with preserved ejection fraction: association with baseline disease severity and subsequent outcome.. JAMA Cardiol.

[6] Kanisicak O, Khalil H, Ivey MJ, Karch J, Maliken BD, Correll RN, Brody MJ, J Lin SC, Aronow BJ, Tallquist MD (2016). Genetic lineage tracing defines myofibroblast origin and function in the injured heart.. Nat Commun.

[7] Tallquist MD, Molkentin JD (2017). Redefining the identity of cardiac fibroblasts.. Nat Rev Cardiol.

[8] Travers JG, Kamal FA, Robbins J, Yutzey KE, Blaxall BC (2016). Cardiac fibrosis: the fibroblast awakens.. Circ Res.

[9] Meng XM, Nikolic-Paterson DJ, Lan HY (2016). TGF-β: the master regulator of fibrosis.. Nat Rev Nephrol.

[10] Shull MM, Ormsby I, Kier AB, Pawlowski S, Diebold RJ, Yin M, Allen R, Sidman C, Proetzel G, Calvin D (1992). Targeted disruption of the mouse transforming growth factor-beta 1 gene results in multifocal inflammatory disease.. Nature.

[11] Bierie B, Chung CH, Parker JS, Stover DG, Cheng N, Chytil A, Aakre M, Shyr Y, Moses HL (2009). Abrogation of TGF-beta signaling enhances chemokine production and correlates with prognosis in human breast cancer.. J Clin Invest.

[12] Inman GJ, Nicolás FJ, Hill CS (2002). Nucleocytoplasmic shuttling of Smads 2, 3, and 4 permits sensing of TGF-beta receptor activity.. Mol Cell.

[13] Schafer S, Viswanathan S, Widjaja AA, Lim WW, Moreno-Moral A, DeLaughter DM, Ng B, Patone G, Chow K, Khin E (2017). IL-11 is a crucial determinant of cardiovascular fibrosis.. Nature.

[14] Ingolia NT, Ghaemmaghami S, Newman JR, Weissman JS (2009). Genome-wide analysis *in vivo* of translation with nucleotide resolution using ribosome profiling.. Science.

[15] Chothani S, Adami E, Viswanathan S, Hubner N, Cook S, Schafer S, Rackham O (2017). Reliable detection of translational regulation with Ribo-seq.. bioRxiv.

[16] Wang M, Sips P, Khin E, Rotival M, Sun X, Ahmed R, Widjaja AA, Schafer S, Yusoff P, Choksi PK (2016). Wars2 is a determinant of angiogenesis.. Nat Commun.

[17] van Heesch S, Witte F, Schneider-Lunitz V, Schulz JF, Adami E, Faber AB, Kirchner M, Maatz H, Blachut S, Sandmann C-L (2019). The translational landscape of the human heart.. Cell.

[18] Heinig M, Adriaens ME, Schafer S, van Deutekom HWM, Lodder EM, Ware JS, Schneider V, Felkin LE, Creemers EE, Meder B (2017). Natural genetic variation of the cardiac transcriptome in non-diseased donors and patients with dilated cardiomyopathy.. Genome Biol.

[19] Schafer S, Adami E, Heinig M, Rodrigues KEC, Kreuchwig F, Silhavy J, van Heesch S, Simaite D, Rajewsky N, Cuppen E (2015). Translational regulation shapes the molecular landscape of complex disease phenotypes.. Nat Commun.

[20] Bolger AM, Lohse M, Usadel B (2014). Trimmomatic: a flexible trimmer for Illumina sequence data.. Bioinformatics.

[21] Langmead B, Trapnell C, Pop M, Salzberg SL (2009). Ultrafast and memory-efficient alignment of short DNA sequences to the human genome.. Genome Biol.

[22] The RNAcentral Consortium (2017). RNAcentral: a comprehensive database of non-coding RNA sequences.. Nucleic Acids Res.

[23] Dobin A, Davis CA, Schlesinger F, Drenkow J, Zaleski C, Jha S, Batut P, Chaisson M, Gingeras TR (2013). STAR: ultrafast universal RNA-seq aligner.. Bioinformatics.

[24] Liao Y, Smyth GK, Shi W (2014). featureCounts: an efficient general purpose program for assigning sequence reads to genomic features.. Bioinformatics.

[25] Calviello L, Mukherjee N, Wyler E, Zauber H, Hirsekorn A, Selbach M, Landthaler M, Obermayer B, Ohler U (2016). Detecting actively translated open reading frames in ribosome profiling data.. Nat Methods.

[26] Love MI, Huber W, Anders S (2014). Moderated estimation of fold change and dispersion for RNA-seq data with DESeq2.. Genome Biol.

[27] Alexa A, Rahnenfuhrer J topGO: Enrichment Analysis for Gene Ontology. R package version 2.36.0..

[28] Tenenbaum D (2019). KEGGREST: Client-side REST access to KEGG. R package version 1.24.0..

[29] Hu B, Yang YT, Huang Y, Zhu Y, Lu ZJ (2017). POSTAR: a platform for exploring post-transcriptional regulation coordinated by RNA-binding proteins.. Nucleic Acids Res.

[30] Shannon P, Markiel A, Ozier O, Baliga NS, Wang JT, Ramage D, Amin N, Schwikowski B, Ideker T (2003). Cytoscape: a software environment for integrated models of biomolecular interaction networks.. Genome Res.

[31] Gabbiani G (2003). The myofibroblast in wound healing and fibrocontractive diseases.. J Pathol.

[32] Sonenberg N, Hinnebusch AG (2009). Regulation of translation initiation in eukaryotes: mechanisms and biological targets.. Cell.

[33] Guo W, Schafer S, Greaser ML, Radke MH, Liss M, Govindarajan T, Maatz H, Schulz H, Li S, Parrish AM (2012). RBM20, a gene for hereditary cardiomyopathy, regulates titin splicing.. Nat Med.

[34] de Bruin RG, Rabelink TJ, van Zonneveld AJ, van der Veer EP (2017). Emerging roles for RNA-binding proteins as effectors and regulators of cardiovascular disease.. Eur Heart J.

[35] Hafner M, Landthaler M, Burger L, Khorshid M, Hausser J, Berninger P, Rothballer A, Ascano M, Jungkamp AC, Munschauer M (2010). Transcriptome-wide identification of RNA-binding protein and microRNA target sites by PAR-CLIP.. Cell.

[36] Van Nostrand EL, Pratt GA, Shishkin AA, Gelboin-Burkhart C, Fang MY, Sundararaman B, Blue SM, Nguyen TB, Surka C, Elkins K (2016). Robust transcriptome-wide discovery of RNA-binding protein binding sites with enhanced CLIP (eCLIP).. Nat Methods.

[37] Park S, Ranjbarvaziri S, Lay FD, Zhao P, Miller MJ, Dhaliwal JS, Huertas-Vazquez A, Wu X, Qiao R, Soffer JM (2018). Genetic regulation of fibroblast activation and proliferation in cardiac fibrosis.. Circulation.

[38] Kanemaru K, Kubota J, Sekiya H, Hirose K, Okubo Y, Iino M (2013). Calcium-dependent N-cadherin up-regulation mediates reactive astrogliosis and neuroprotection after brain injury.. Proc Natl Acad Sci U S A.

[39] Noveroske JK, Lai L, Gaussin V, Northrop JL, Nakamura H, Hirschi KK, Justice MJ (2002). Quaking is essential for blood vessel development.. Genesis.

[40] Zhang Y, Si Y, Ma N, Mei J (2015). The RNA-binding protein PCBP2 inhibits Ang II-induced hypertrophy of cardiomyocytes though promoting GPR56 mRNA degeneration.. Biochem Biophys Res Commun.

[41] Subramaniam D, Ramalingam S, Linehan DC, Dieckgraefe BK, Postier RG, Houchen CW, Jensen RA, Anant S (2011). RNA binding protein CUGBP2/CELF2 mediates curcumin-induced mitotic catastrophe of pancreatic cancer cells.. PLoS One.

[42] Kung YA, Hung CT, Chien KY, Shih SR (2017). Control of the negative IRES trans-acting factor KHSRP by ubiquitination.. Nucleic Acids Res.

[43] Adereth Y, Dammai V, Kose N, Li R, Hsu T (2005). RNA-dependent integrin alpha3 protein localization regulated by the Muscleblind-like protein MLP1.. Nat Cell Biol.

[44] Yang R, Gaidamakov SA, Xie J, Lee J, Martino L, Kozlov G, Crawford AK, Russo AN, Conte MR, Gehring K (2011). La-related protein 4 binds poly(A), interacts with the poly(A)-binding protein MLLE domain via a variant PAM2w motif, and can promote mRNA stability.. Mol Cell Biol.

[45] Conn SJ, Pillman KA, Toubia J, Conn VM, Salmanidis M, Phillips CA, Roslan S, Schreiber AW, Gregory PA, Goodall GJ (2015). The RNA binding protein quaking regulates formation of circRNAs.. Cell.

[46] Wang ET, Cody NA, Jog S, Biancolella M, Wang TT, Treacy DJ, Luo S, Schroth GP, Housman DE, Reddy S (2012). Transcriptome-wide regulation of pre-mRNA splicing and mRNA localization by muscleblind proteins.. Cell.

[47] Glisovic T, Bachorik JL, Yong J, Dreyfuss G (2008). RNA-binding proteins and post-transcriptional gene regulation.. FEBS Lett.

[48] Burstein B, Libby E, Calderone A, Nattel S (2008). Differential behaviors of atrial versus ventricular fibroblasts: a potential role for platelet-derived growth factor in atrial-ventricular remodeling differences.. Circulation.

[49] Gerstberger S, Hafner M, Tuschl T (2014). A census of human RNA-binding proteins.. Nat Rev Genet.

[50] Schwarz RI (2015). Collagen I and the fibroblast: high protein expression requires a new paradigm of post-transcriptional, feedback regulation.. Biochem Biophys Rep.

