# Supplementary material for: Widespread Translational Control of Fibrosis in the Human Heart by RNA-Binding Proteins
Source: Circulation. 2019 Jul 9;140(11):937–51. doi: 10.1161/CIRCULATIONAHA.119.039596 (PMC6749977; doi:10.1161/CIRCULATIONAHA.119.039596)
Supplement: Supplementary file 1 [file cir-140-937-s001.pdf]

Supplementary Material

Supplementary Figures

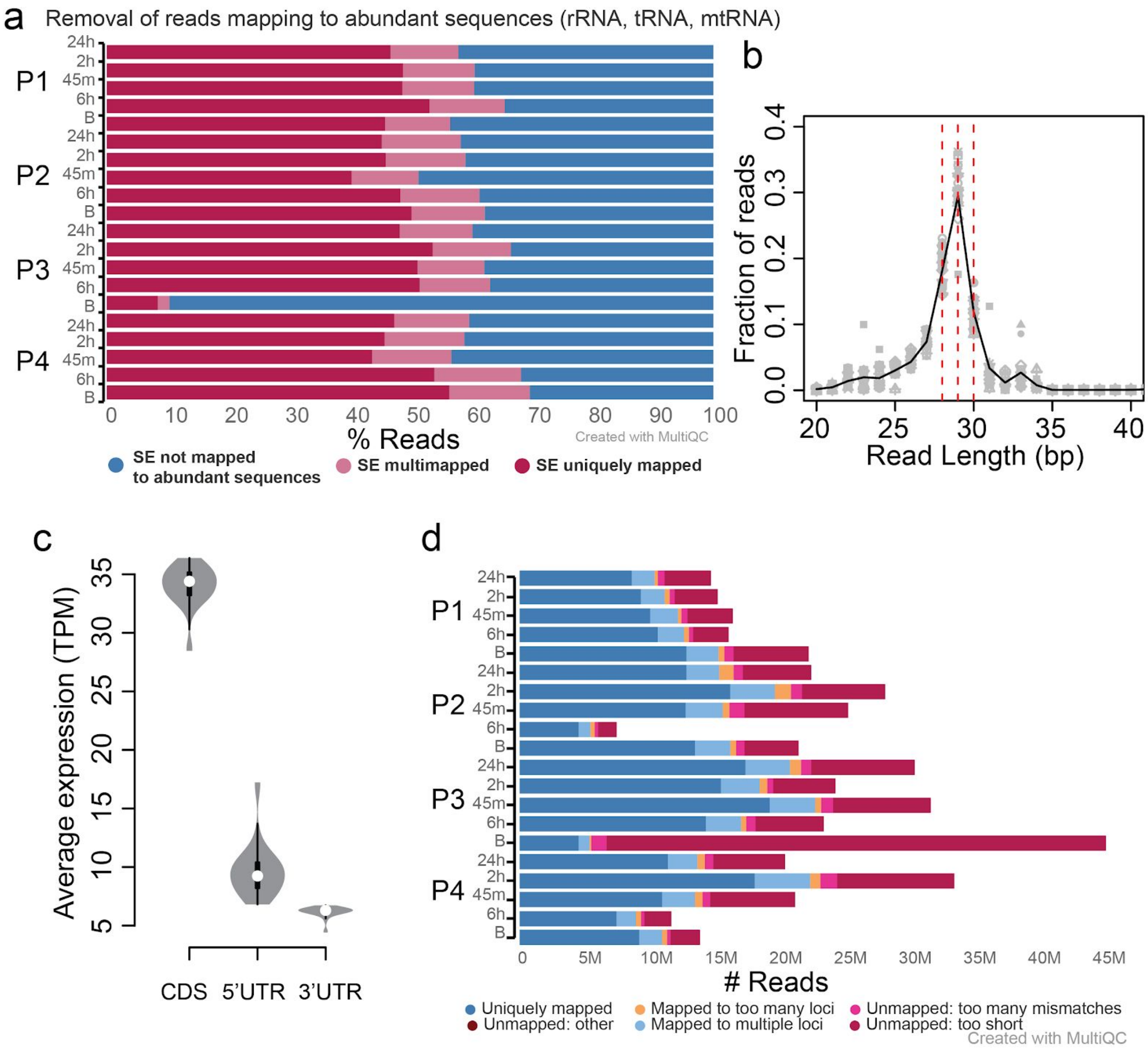

Supplementary Figure 1: a. Percentage of trimmed reads mapping to abundant sequences (ribosomal RNA, transfer RNA, mitochondrial RNA). **b**. Length distribution of reads after removal of abundant sequences showing 28, 29, 30 as the predominant lengths of ribosome protected fragments (RPFs). **c**. Average expression (Transcripts per million mapped reads) in each region of protein coding genes showing higher coverage on coding sequence (CDS) compared to the untranslated regions as expected for RPFs. **d**. Number of reads mapping to the human genome (hg38) per sample using STAR aligner. Quality statistics calculated and visualized using MultiQC[1].

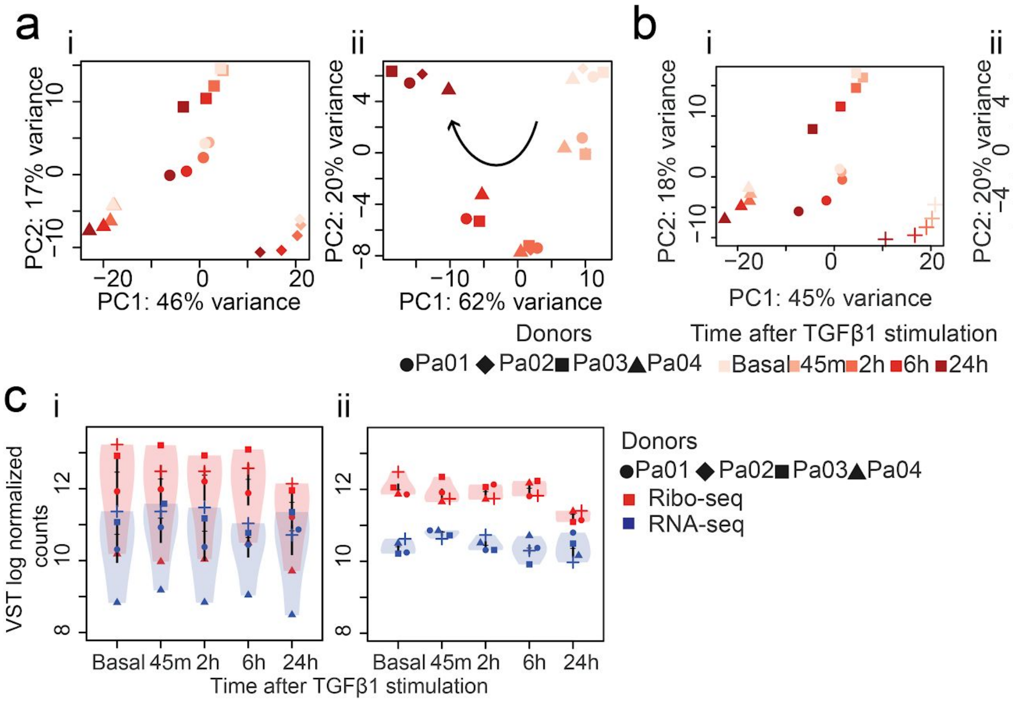

Supplementary Figure 2: a-b. Principal component analysis (top 500 genes, ranked by variance across samples) of the RNA-seq **(a)** and Ribo-seq data **(b)** across the fibrotic response. PC1 accounts for 45-46% of the variance in the gene expression and samples are grouped by patient indicating a patient batch effect (**a: i b: i**). After batch correction, PC1 accounts for 59%-62% of the variance and now separates the time-points (**a: ii, b: ii)**. **c.** Ribo-seq and RNA-seq counts for Integrin 7A exhibit a strong patient effect, not allowing detection as a DTEG **(c: i)**, which is ameliorated after batch correction on normalized VST counts **(c: ii)**. Batch effect removal was carried out using limma package[2] in R.

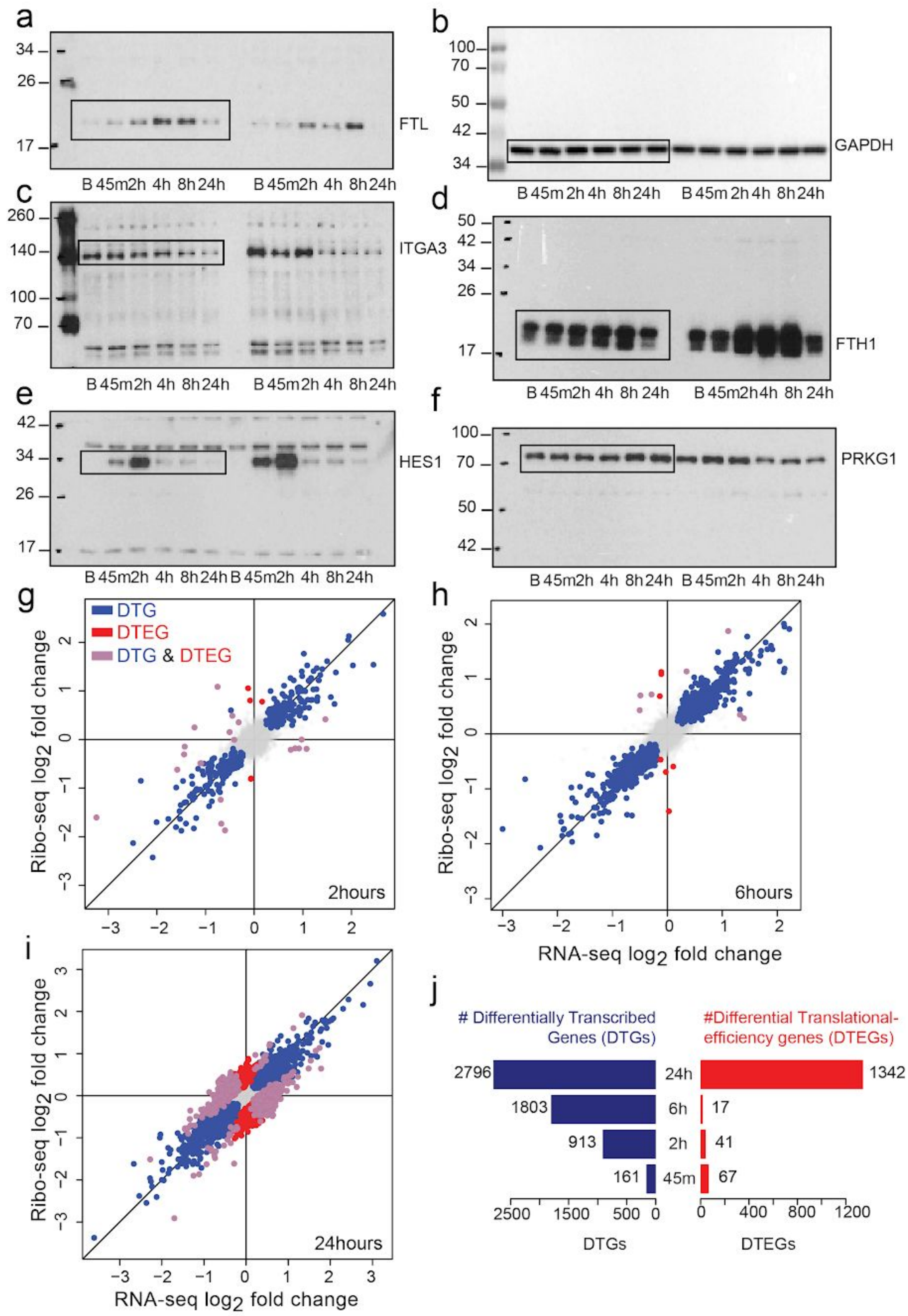

Supplementary Figure 3: a-f: Western blotting of exclusive (FTL (**a**), FTH1 (**c**), ITGA3 (**d**)), intensified (HES1 (**e**)) and buffered (PRKG1 (**f**)) genes with control (GAPDH (**b**)) in two patients at baseline and 4 time points after TGFβ1 stimulation in primary human cardiac fibroblasts. **g-i:** Log-fold changes in the mRNA and ribosome occupancy at 2

hours (**g**), 6 hours (**h**) and 24 hours (**i**) after TGFβ1 stimulation. DTG: Differentially transcribed genes, DTEG: Differential translational-efficiency genes. **j:** DTGs and DTEGs detected at each time point after TGFβ1 stimulation.

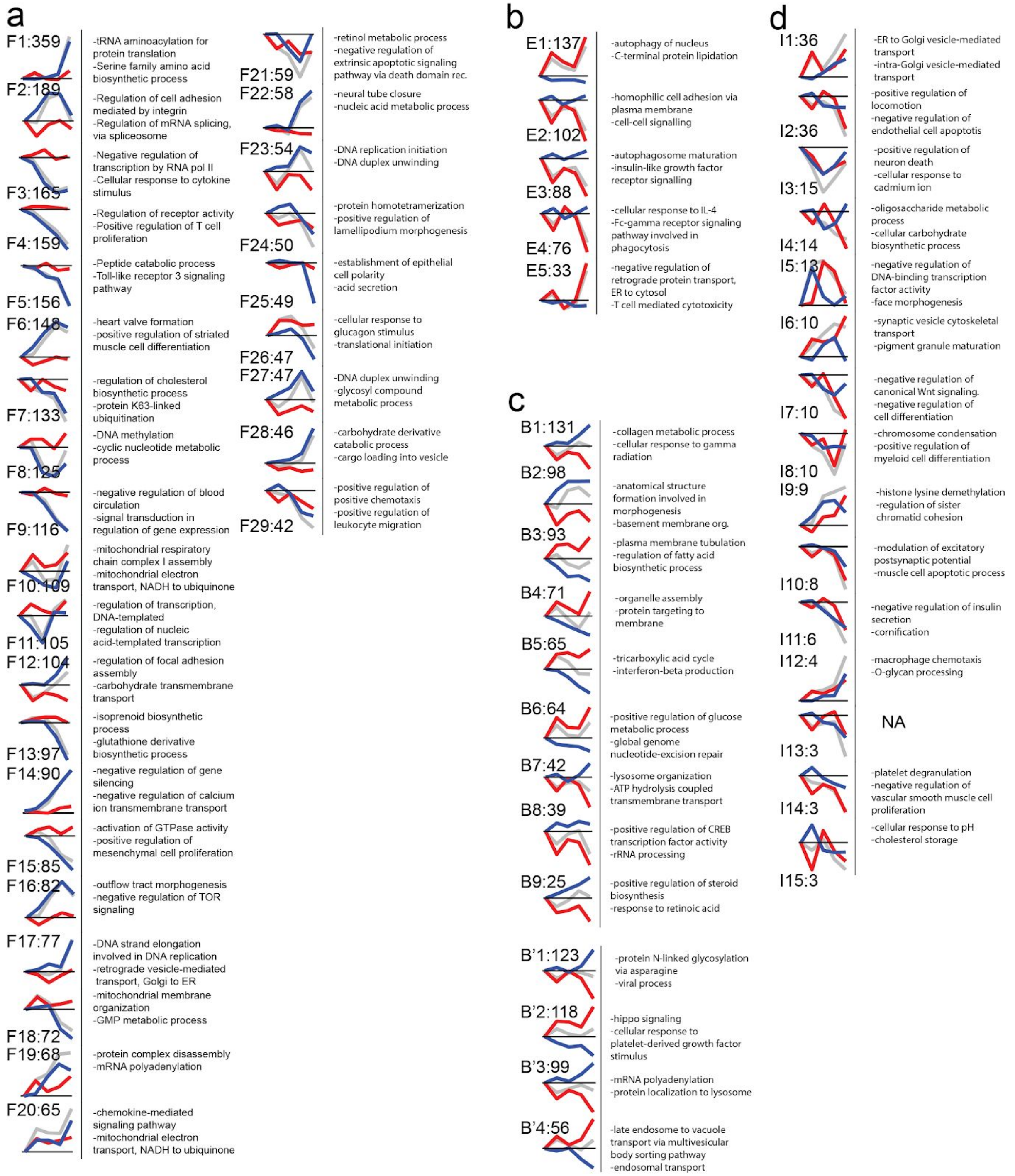

Supplementary Figure 4: Hierarchical clustering of different regulatory groups including **a.** F: forwarded **b.** E: exclusive **c.** B: buffered, B': completely buffered (special case) and **d.** I: intensified at the translational level. Refer Supplementary table 4 for gene regulatory groups. The cluster IDs are represented as (gene regulatory group)(#):(Number of genes in that cluster). For instance, F1:359 is the first forwarded cluster with 359 genes. The clusters are ordered based on cluster size in each regulatory class.

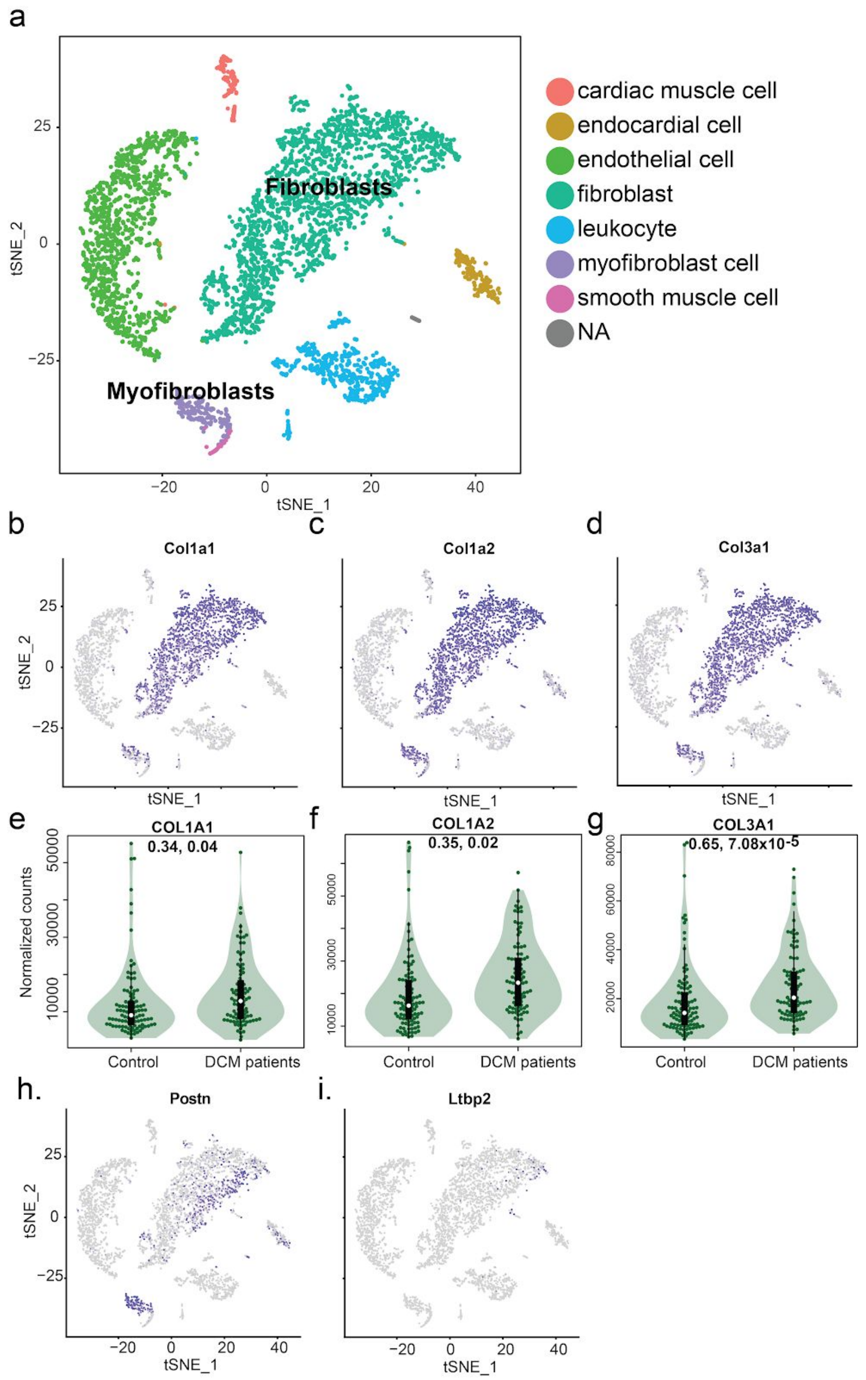

Supplementary figure 5: Cardiac fibrosis markers in adult mouse heart tissue[3] **and end-stage DCM patients**. **a.** t-SNE plot showing fibroblasts as one of the largest cell populations in adult mouse hearts. Expression of **b.** Collagen I alpha 1, Col1a1, **c.** Collagen I alpha 2, Col1a2 and **d.** Collagen III alpha 1, Col3a1 is specific to fibroblasts/myofibroblasts in adult mouse hearts and they are also **e-g.** upregulated in DCM patients (Fold change, FDR). **h-i.** Expression of Periostin, Postn (**h**) and Latent TGFβ binding protein 2, Ltbp2 (**i**) is also specific to fibroblasts/myofibroblasts.

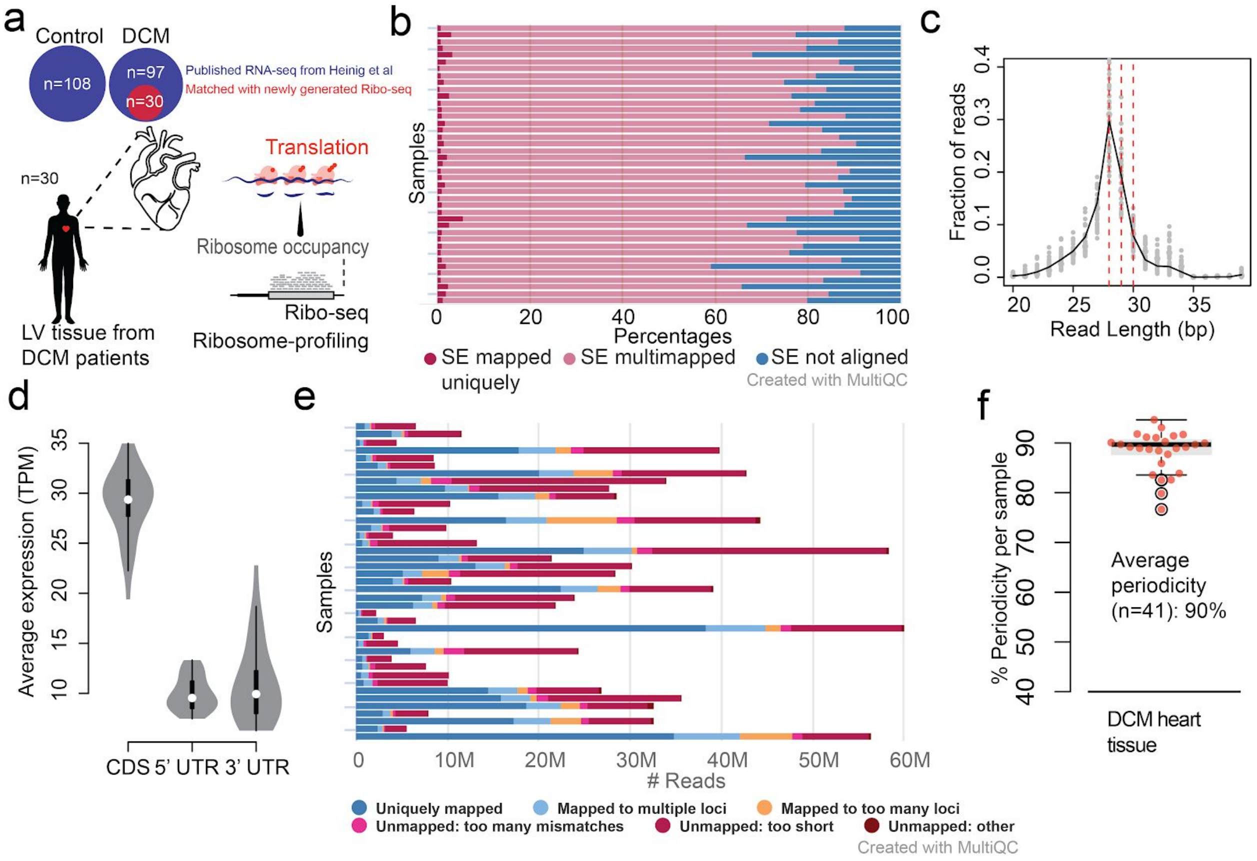

Supplementary Figure 6: Genome-wide translational profiling of dilated cardiomyopathy patients. a. Control (n=108) and end-stage DCM patient (n=97) RNA-seq dataset was obtained from our previous study[4] for differential expression analysis. We generated Ribo-seq libraries (n=30, out of 97 DCM patients) to carry out translational efficiency analysis. **b.** Percentage of trimmed Ribo-seq reads mapping to abundant sequences (rRNA, tRNA, mitochondrial RNA). **c**. Length distribution of reads after removal of abundant sequence mapped reads showing 28, 29, 30 as the predominant lengths of ribosome protected fragments (RPFs). **d**. Average expression (TPM) in each region of protein coding genes showing higher coverage of coding sequence (CDS) as expected for RPFs. **e**. Number of reads mapping to the human genome (hg38) per sample. **f.** Average periodicity of 90% across all 30 samples.

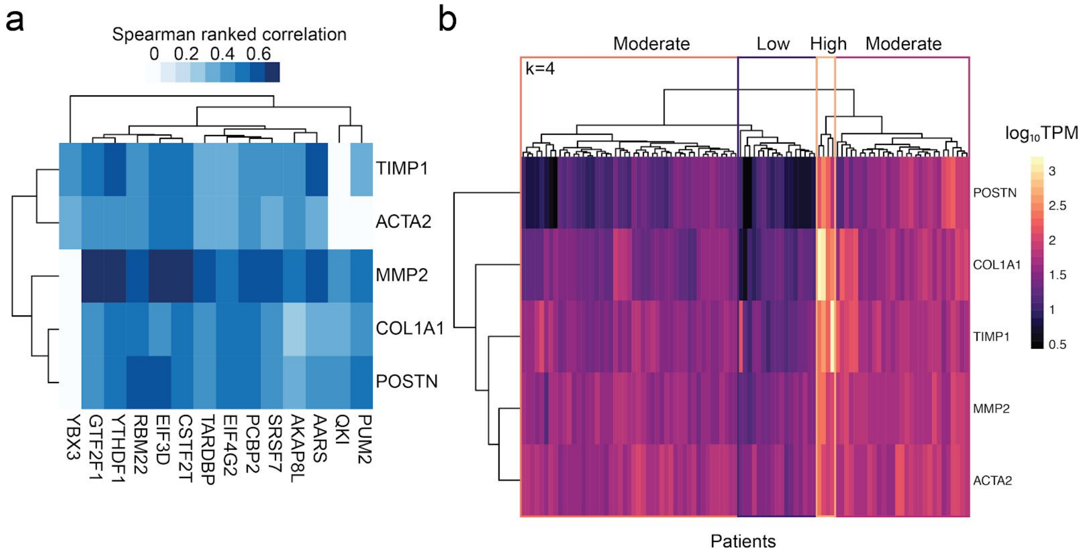

Supplementary Figure 7: a. Gene expression correlation (spearman ranked) of RNA-binding proteins with fibrosis markers. Only significant correlations based on p-value < 0.05 are shown. **b.** Hierarchical clustering to stratify patients based on fibrosis marker gene expression. Tree was cut into four clusters, resulting in four groups of fibrogenic marker expression severity.

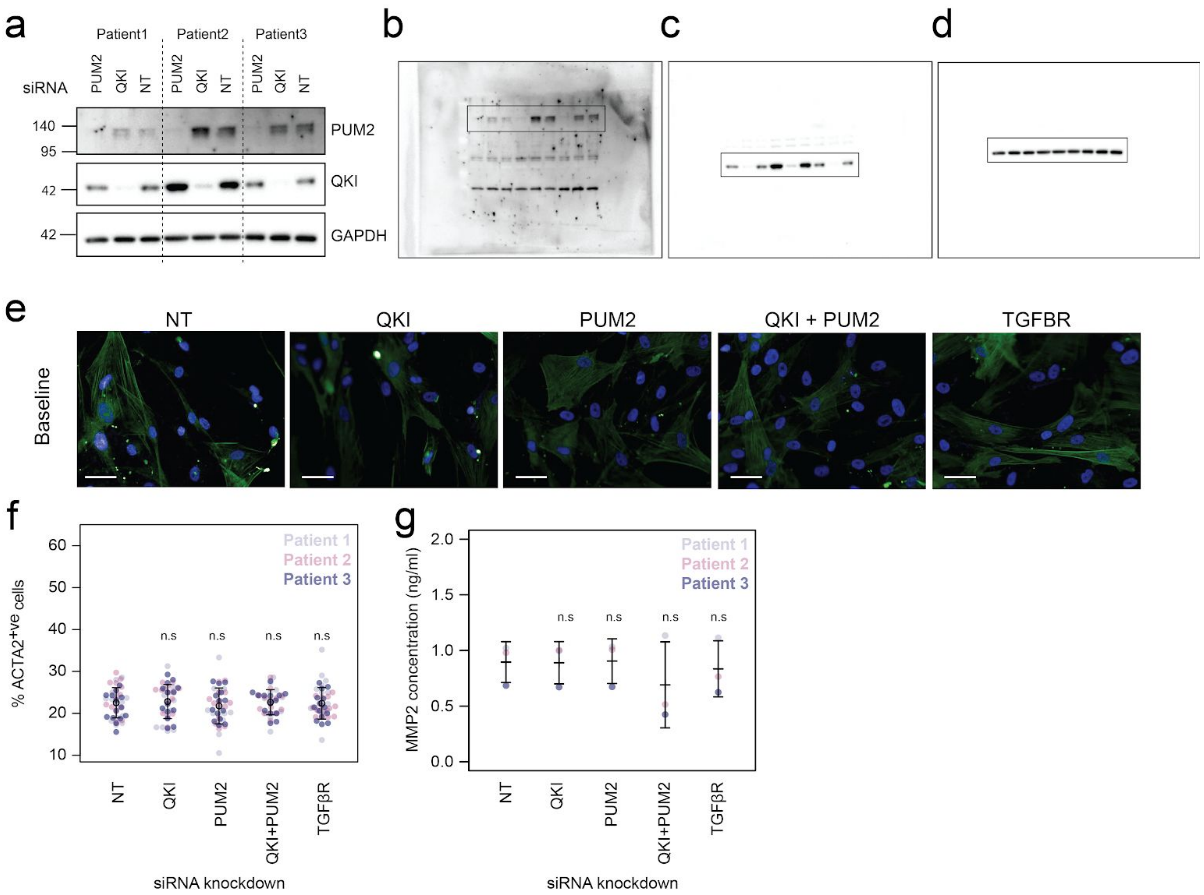

Supplementary figure 8: Knockdown of Quaking protein (QKI) and Pumilio RNA Binding Family Member 2 (PUM2) in baseline fibroblasts from three cardiac patients. NT: Non-targeting control siRNA. **a.** Western blot showing successful knockdown of QKI and PUM2 in all three patients. Full blots shown in **b-d.** for PUM2 (b), QKI (c) and loading control (d). **e.** Microscopic images show fibroblasts with immunostaining for α-smooth muscle actin (ACTA2). Scale bar equals 50μm. **f.** Fluorescence quantified on the Operetta high-content imaging platform after immunostaining for ACTA2 (28 measurements across four wells) and normalized for cell count. **g.** Total concentration of MMP2 in the supernatant (n=3, biologically independent samples) was quantified by ELISA. P-values were determined by one-way ANOVA and corrected for comparisons to the same sample (NT) using Dunnett's test. n.s. not significant.

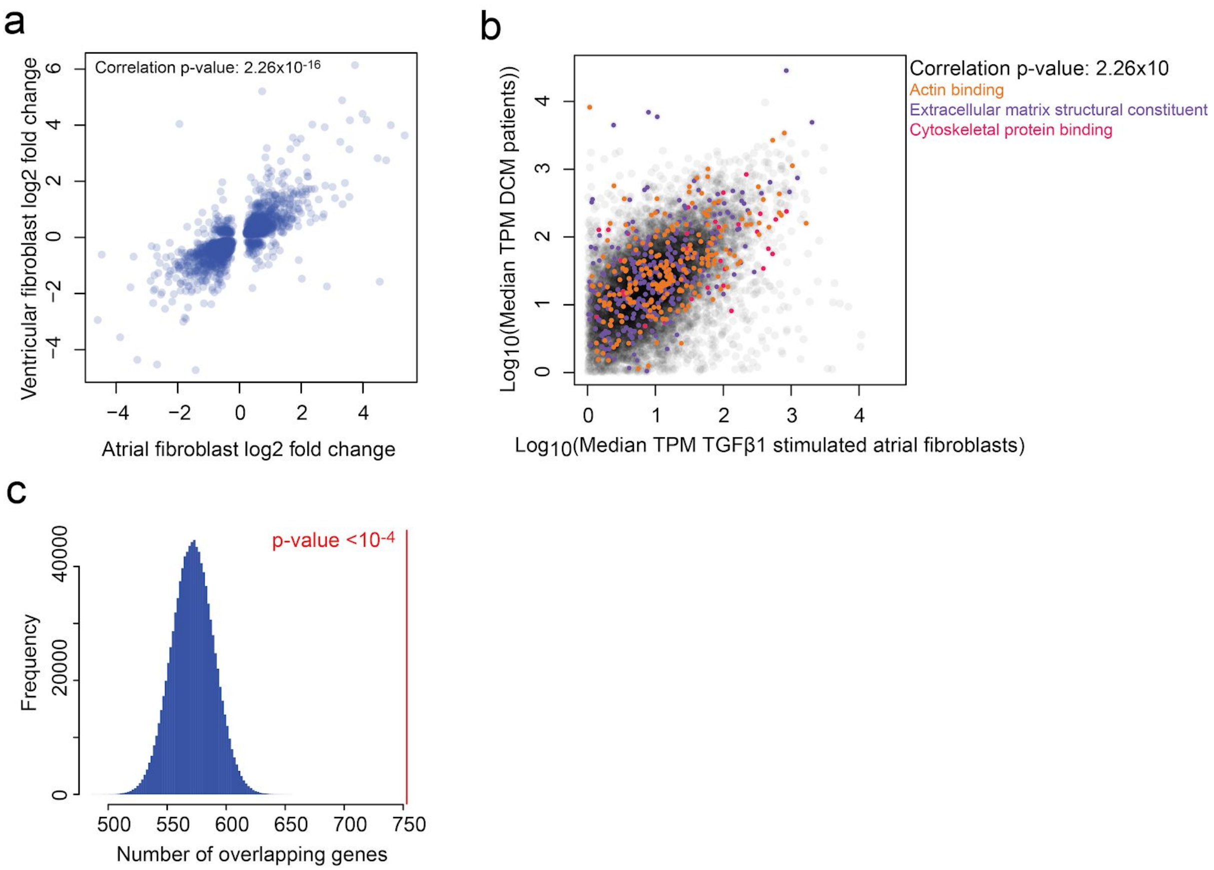

Supplementary figure 9: Common pro-fibrotic response in different datasets of fibrotic disease. a. Correlation of fold changes after TGFβ1 stimulation in atrial and ventricular fibroblasts. **b.** Correlation of expression (logTPM) in TGFβ1 stimulated atrial fibroblasts and end-stage DCM patients. Genes in GO terms like actin binding, ECM structural constituent and cytoskeletal protein binding highlighted. **c.** Significant overlap of genes dysregulated in TGFβ1 stimulated atrial fibroblasts and DCM patients. Blue: Background distribution of random overlap. Red: True overlap.

Supplementary Tables

Supplementary table 1: Patient information for four male chinese individuals undergoing coronary artery bypass grafting

Patient	Age
P1	59
P2	52
P3	57
P4	51

Supplementary table 2: Antibody information for Immunostaining, Operetta imaging, Confocal microscopy and Western Blotting

Antibody	Catalogue number	Company	Experiment
ACTA2	ab7817	Abcam	Operetta imaging and immunostaining confocal microscopy
Collagen I	ab34710	Abcam	Operetta imaging
POSTN	ab14041	Abcam	Operetta imaging
Goat anti-rabbit Alexa Flour-488	150017	Abcam	Operetta imaging
Goat anti-mouse Alexa Flour-488	150113	Abcam	Operetta imaging
Goat anti-mouse Alexa Fluor-555	A-21424	Thermo Fisher	Immunostaining confocal microscopy
p-ERK1/2	4370	CST	Western blotting
ERK1/2	4695	CST	Western blotting
FTL	ab109373	Abcam	Western blotting
FTH1	4393	CST	Western blotting
GAPDH	2118	CST	Western blotting
HES	11988	CST	Western blotting
ITGA3	sc-374242	SantaCruz	Western blotting
PKG-1	3248	CST	Western blotting
p-SMAD2	5339	CST	Western blotting
SMAD2	3108	CST	Western blotting
QKI	A300-183A	Bethyl Labs	Western blotting
PUM2	A300-202A	Bethyl Labs	Western blotting

Supplementary table 3: Software and tools used for data analysis

Purpose	Tool	Version	Command
Demultiplexing	bcl2fastq	*V2.19.0.316*	bcl2fastq --no-lane-splitting
Adaptor trimming	Trimmomatic	*V0.36*	**Ribo-seq**: java -jar trimmomatic-0.36.jar SE -phred33 infile outfile ILLUMINACLIP:All_TruSeqForTrimmomatic.fa:2:30:10 MAXINFO:20:0.5 MINLEN:20<br>**RNA-seq**: java -jar trimmomatic-0.36.jar PE -phred33 infiles outfiles ILLUMINACLIP:All_TruSeqForTrimmomatic.fa:2:30:10 MAXINFO:35:0.5 MINLEN:35
Read clipping	Fastx toolkit	*V0.0.14*	fastx_trimmer -l 29 -Q33 -z
Abundant sequence removal	Bowtie2	*V2.2.9*	bowtie2 -L 20 -x human_abundant_combine
Genome alignment	STAR	*020201*	STAR --alignSJDBoverhangMin 1 --alignSJoverhangMin 51 --outFilterMismatchNmax 2 --alignEndsType EndToEnd
Gene expression quantification	Feature Counts	*1.5.1*	featureCounts -t CDS -g gene_id -O -s 1 -J -R -G Homo_sapiens.GRCh38.dna.primary_assembly.fa -a GTF
DTEG identification	DESeq2	*1.18.1*	Design =~ Patient + Time + Sequencing + Time:Sequencing<br><br>https://github.com/SGDDNB/DTG-detection/blob/master/getDTG.md
Batch effect removal	Limma[5]	*3.34.9*	removeBatchEffect(counts, batch)
Heatmap for periodicity	pheatmap[6]	*1.0.8*	pheatmap(data)
Clustering	Hclust ward.D[7]<br>cutreeDynamic[8]	 *1.63-1*	hclust(data, method=ward.D)

Supplementary table 4: Classification of genes into regulation categories based on significance and fold change relationship. s: Significant changes calculated using DESeq2 with FDR<0.05; n.s: Not significant. FC: Fold change. Concordant: Significant fold changes are in the same direction. Counteracting: Significant fold changes are in the opposite direction. Schematic: ΔRPF: gray, ΔRNA: Blue, ΔTE: Red.

Category	RPF	RNA	ΔTE	FC relationship	DTG/DTEG	Schematic
Forwarded	s	s	n.s.	RPF concordant with RNA	DTG	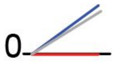
Exclusive	s	n.s.	s	RPF changing independent of RNA	DTEG	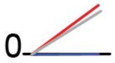
Buffered (special case)	n.s.	s	s	ΔTE counteracting RNA; RPF FC = 0	DTG & DTEG	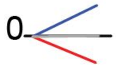
Buffered	s	s	s	ΔTE counteracting RNA	DTG & DTEG	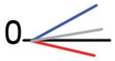
Intensified	s	s	s	ΔTE intensifying RNA	DTG & DTEG	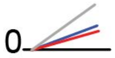

Supplementary Files

File 1: Log-fold changes and statistics for all protein-coding genes (Ensembl and Refseq)
File 2: Genes in regulatory category: Buffered, Translation exclusive, Forwarded, Intensified
File 3: Gene Ontology enrichment in gene regulatory groups
File 4: Gene Ontology enrichment in transient gene clusters
File 5: RNA-binding proteins and their target correlations in dilated cardiomyopathy patients
File 6: KEGG pathway analysis of each RBPs target network

Supplementary References

1. Ewels P, Magnusson M, Lundin S, Käller M. MultiQC: summarize analysis results for multiple tools and samples in a single report. *Bioinformatics*. 2016;32:3047–3048.

2. Ritchie ME, Phipson B, Wu D, Hu Y, Law CW, Shi W, Smyth GK. limma powers differential expression analyses for RNA-sequencing and microarray studies. *Nucleic Acids Res*. 2015;43:e47.

3. Tabula Muris Consortium, Overall coordination, Logistical coordination, Organ collection and processing, Library preparation and sequencing, Computational data analysis, Cell type annotation, Writing group, Supplemental text writing group, Principal investigators. Single-cell transcriptomics of 20 mouse organs creates a Tabula Muris. *Nature*. 2018;562:367–372.

4. Heinig M, Adriaens ME, Schafer S, van Deutekom HWM, Lodder EM, Ware JS, Schneider V, Felkin LE, Creemers EE, Meder B, Katus HA, Rühle F, Stoll M, Cambien F, Villard E, Charron P, Varro A, Bishopric NH, George AL Jr, Dos Remedios C, Moreno-Moral A, Pesce F, Bauerfeind A, Rüschendorf F, Rintisch C, Petretto E, Barton PJ, Cook SA, Pinto YM, Bezzina CR, Hubner N. Natural genetic variation of the cardiac transcriptome in non-diseased donors and patients with dilated cardiomyopathy. *Genome Biol*. 2017;18:170.

5. Ritchie ME, Phipson B, Wu D, Hu Y, Law CW, Shi W, Smyth GK. limma powers differential expression analyses for RNA-sequencing and microarray studies. *Nucleic Acids Res*. 2015;43:e47.

6. Raivo Kolde (2015). pheatmap: Pretty Heatmaps. R package version 1.0.8.

7. Murtagh F, Legendre P. Ward's Hierarchical Agglomerative Clustering Method: Which Algorithms Implement Ward's Criterion? *J Classification*. 2014;31:274–295.

8. Langfelder P, Zhang B, Horvath S. Defining clusters from a hierarchical cluster tree: the Dynamic Tree Cut package for R. Bioinformatics. 2008;24:719–720.
